# Crystal structures of a series of 6-aryl-1,3-diphenyl­fulvenes

**DOI:** 10.1107/S205698901900700X

**Published:** 2019-05-21

**Authors:** Andrew J. Peloquin, Sonya K. Adas, Scott T. Iacono, Gary J. Balaich

**Affiliations:** a Department of Chemistry & Chemistry Research Center, United States Air Force Academy, Colorado Springs, CO 80840, USA

**Keywords:** crystal structure, fulvene, methyl­ation patterns, C—H⋯π ring inter­actions

## Abstract

The synthesis and crystal structures of a series of 6-aryl­fuvlenes (fulvene is 5-methyl­idene­cyclo­penta-1,3-diene) with varying methyl­ation patterns on the 6-phenyl substituent are reported, namely 6-(3-methyl­phen­yl)-1,3-di­phenyl­fulvene (C_25_H_20_), 6-(4-methyl­phen­yl)-1,3-di­phenyl­fulvene (C_25_H_20_), 6-mesityl-3-di­phenyl­fulvene (C_27_H_24_) and 6-(2,3,4,5,6-penta­methyl­phen­yl)-1,3-di­phenyl­fulvene (C_29_H_28_). The bond lengths are typical of those observed in related fulvenes. A network of C—H⋯π ring inter­actions consolidates the packing in each structure.

## Abstract 2

The synthesis and structures of a series of 6-aryl-1,3-diphenyl­fuvlenes with (fulvene is 5-methyl­idene­cyclo­penta-1,3-diene) varying methyl­ation patterns on the 6-phenyl substituent are reported. A network of C—H⋯π ring inter­actions consolidates the packing in each structure.

## Chemical context   

**Figure 1:**
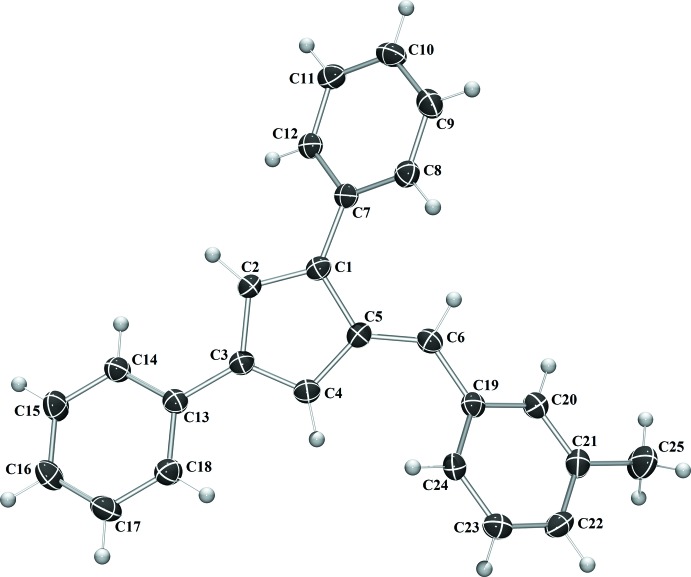
The mol­ecular structure of **I**. Displacement ellipsoids are shown at the 50% probability level.

Penta­fulvenes have garnered inter­ested because of their unique cross-conjugated electronic system. Fulvenes have been explored for applications as chromophores (Jayamurugan *et al.*, 2013 ▸), frustrated Lewis pair scaffolds (Mömming *et al.*, 2011 ▸), and ligands for metal–fulvene complexes (Erker, 2011 ▸). To facilitate the inclusion in transition-metal complexes, reduction to a cyclo­penta­diene ligand (Gómez-Ruiz *et al.*, 2005 ▸) or reductive coupling to ansa bis-cyclo­penta­diene ligand (Adas & Balaich, 2018 ▸) are the most common reactions. As part of our work in this area, we now report the syntheses and crystal structures of a series of 1,3-di­phenyl­fulvenes bearing 6-phenyl substituent with diverse methyl­ation patterns, *viz*. 1,3-diphenyl-6-(3-methyl­phen­yl)fulvene (C_25_H_20_) **I**, 1,3-diphenyl-6-(4-methyl­phen­yl)fulvene (C_25_H_20_) **II**, 3-diphenyl-6-mesitylfulvene (C_27_H_24_) **III** and 1,3-diphenyl-6-(2,3,4,5,6-penta­methyl­phen­yl)fulvene (C_29_H_28_) **IV** (Figs. 1 ▸–4 ▸ ▸ ▸).
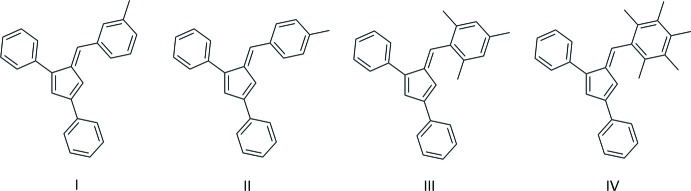


## Structural commentary   

Compounds **I** and **IV** crystallize in the monoclinic space group *C*2/*c* (Fig. 1 ▸), compound **II** in the monoclinic space group *P*2_1_/*c*, and compound **III** the ortho­rhom­bic space group *Pca*2_1_. With the exception of **III**, in which the asymmetric unit contains two complete fulvene mol­ecules, each compound crystallizes with one mol­ecule per asymmetric unit. In each compound, the expected alternating long–short intra-ring bond lengths are observed. The phenyl substituents are rotated from 19.50 (6) to 64.15 (7)° from the cyclo­penta­diene core of the fulvene (Table 1 ▸). The rotation is larger for each substituent in **IV**, likely because of the additional steric inter­actions provided by the penta­methyl substituent.

For fulvene **I**, the phenyl substituents are rotated 32.08 (7), 19.50 (6) and 31.99 (6)° from the cyclo­penta­diene core for the 1-phenyl, 3-phenyl, and 6-phenyl substituents, respectively. In compound **II**, the phenyl substituents are rotated 31.83 (5), 20.92 (5) and 35.13 (5)° from the cyclo­penta­diene core for the 1-phenyl, 3-phenyl, and 6-phenyl substituents, respectively. For compound **III**, the phenyl substituents are rotated an average of 21.33 (13), 38.02 (13) and 57.22 (14)° from the cyclo­penta­diene core for the 1-phenyl, 3-phenyl, and 6-phenyl substituents, respectively. In fulvene **IV**, each phenyl ring is rotated further from the core of the fulvene mol­ecule, likely because of the additional steric inter­actions provided by the penta­methyl­phenyl substituent. The phenyl substituents are rotated by 41.65 (7), 25.17 (7) and 64.15 (7)° from the cyclo­penta­diene core for the 1-phenyl, 3-phenyl, and 6-phenyl substituents, respectively.

## Supra­molecular features   

**Figure 5:**
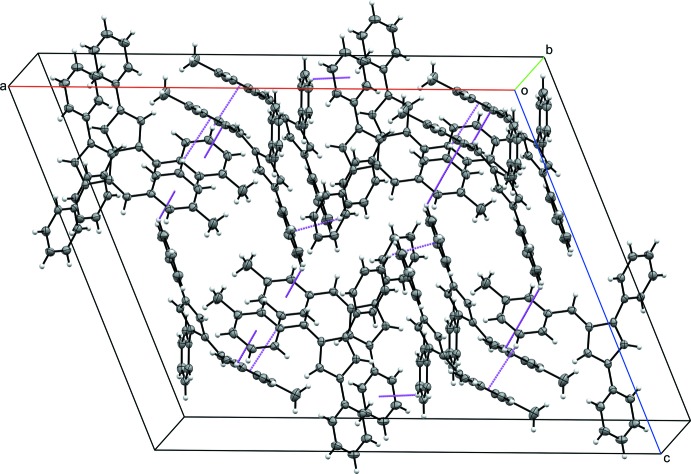
The crystal packing of **I**. Displacement ellipsoids are shown at the 50% probability level. C—H⋯π ring inter­actions (Table 2 ▸) are shown as dashed lines.

**Figure 6:**
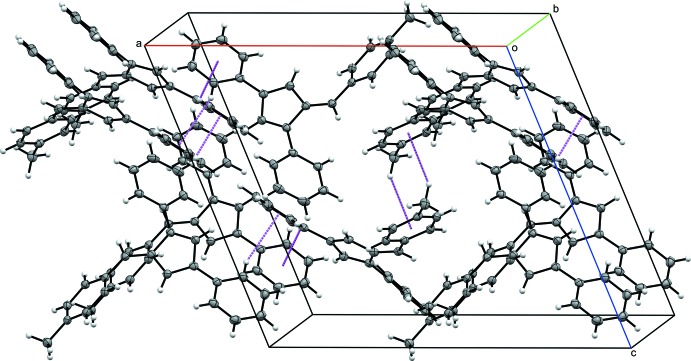
The crystal packing of **II**. Displacement ellipsoids are shown at the 50% probability level. C—H⋯π ring inter­actions (Table 3 ▸) are shown as dashed lines.

**Figure 7:**
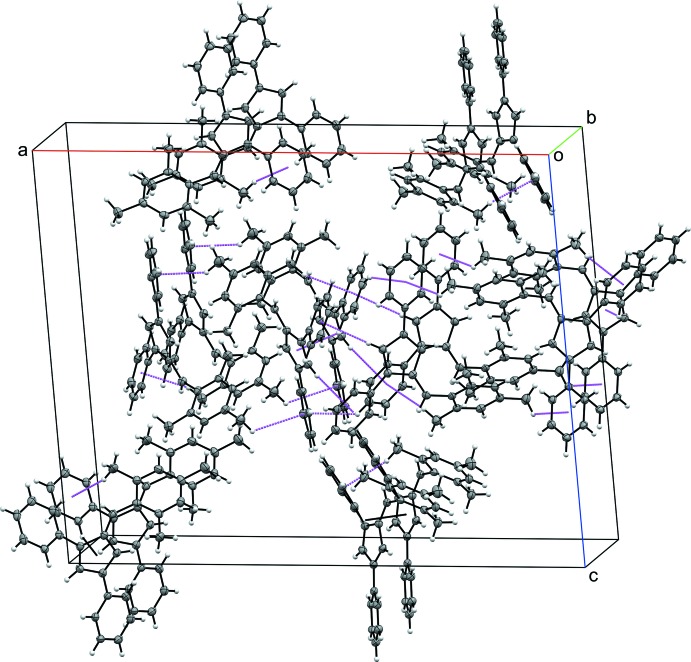
The crystal packing of **III**. Displacement ellipsoids are shown at the 50% probability level. C—H⋯π ring inter­actions (Table 4 ▸) are shown as dashed lines.

**Figure 8:**
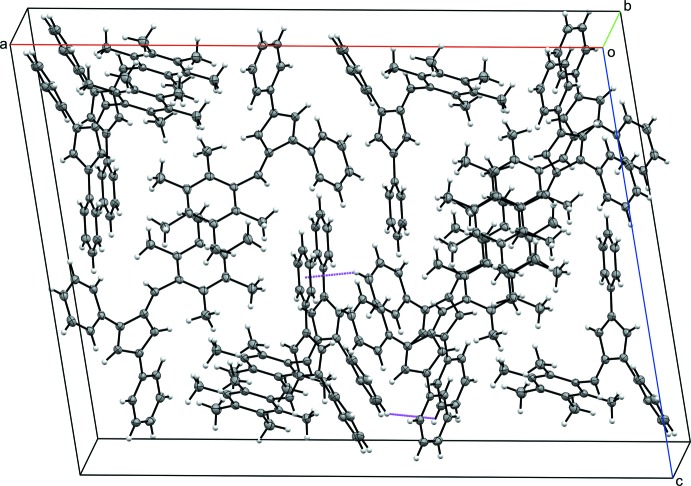
The crystal packing of **IV**, viewed along the *b* axis. Displacement ellipsoids are shown at the 50% probability level. C—H⋯π ring inter­actions (Table 5 ▸) are shown as dashed lines.

The packing for each compound **I**–**IV** is consolidated through a series of C—H⋯π ring inter­actions. In **I**, each mol­ecule participates in C—H⋯π ring inter­actions with six other fulvene mol­ecules. Each mol­ecule acts as a C—H donor through the hydrogen atoms in the *para* position of each phenyl substituent, H10 and H16, as well as a *meta* hydrogen atom, H23, from the 6-(3-methyl­phen­yl) substituent. Additionally, the π ring of the 3-phenyl and 6-(3-methyl­phen­yl) substituents accept C—H inter­actions, with the latter accepting donations from both sides of the ring (Table 2 ▸ and Fig. 5 ▸). In the crystal structure of **II**, each mol­ecule inter­acts with five other fulvene mol­ecules. Phenyl hydrogen atoms H12 and H17 as well as methyl hydrogen atom H25*A* act as donors. The 3-phenyl and 6-(4-methyl­phen­yl) substituents act as C—H acceptors, with the former accepting donations from both sides of the ring (Table 3 ▸ and Fig. 6 ▸). The inter­actions differ between the two mol­ecules within the asymmetric unit of **III**. One of the mol­ecules contributes four C—H donor sites, H37, H39, H53*A*, and H54*C*, with the π ring of each phenyl substituent as well as the fulvene core acting as acceptors. In the other mol­ecule, H10 and H25*C* act as C—H donors with the π system of the 1-phenyl and 3-phenyl substituents accepting, the latter accepting C—H inter­actions from both sides of the ring (Table 4 ▸ and Fig. 7 ▸). Fulvene **IV** inter­acts with four other mol­ecules *via* C—H⋯π ring inter­actions. The *para* hydrogen atom of the 1-phenyl substituent and one of the hydrogen atoms of the *para* methyl group of the 6-(2,3,4,5,6-penta­metyhlphen­yl) substituent, H27*C*, serve as C—H donors for two separate fulvene mol­ecules. The 6-(2,3,4,5,6-penta­metyhlphen­yl) π ring accepts C—H donation from two additional mol­ecules (Table 5 ▸ and Fig. 8 ▸).

## Database survey   

A survey of the November 2019 release of the Cambridge Structure Database (Groom *et al.*, 2016 ▸), with updates through February 2019, was made using the program *Mogul* (Bruno *et al.*, 2004 ▸). A search for 1,3-diphenyl fulvenes and 6-aryl-1,3-diphenyl fulvenes yielded 78 and 35 results, respectively. In both cases, the phen­yl–fulvene torsion angles produce a bimodal distribution with broad peaks at 50 and 130°. The torsion angles in **I**–**IV** are therefore not unusual.

## Synthesis and crystallization   

Each compound was prepared by a modified literature procedure (Peloquin *et al.*, 2012 ▸).

**1,3-diphenyl-6-(3-methyl­phen­yl)fulvene (I).** To a vigorously stirred solution of 1,3-di­phenyl­cyclo­penta­diene (0.230 g, 1.05 mmol) in absolute EtOH (25 ml), 3-methyl­benzaldehyde (0.189 g, 1.58 mmol) and pyrrolidine (0.12 g, 1.68 mmol) were added. The reaction mixture was allowed to stir at room temperature for 22 h. The precipitate from the reaction mixture was vacuum filtered, washed with cold absolute EtOH (3 × 30 ml), and vacuum dried to give **I** as a dark-red solid (0.211 g, 63%). Red prisms suitable for single-crystal X-ray diffraction were obtained from diethyl ether solution by slow evaporation.

**1,3-diphenyl-6-(4-methyl­phen­yl)fulvene (II).** To a vigorously stirred solution of 1,3-di­phenyl­cyclo­penta­diene (0.336 g, 1.42 mmol) in absolute EtOH (8 ml), 4-methyl­benzaldehyde (0.25 ml, 2.13 mmol) and pyrrolidine (0.14 ml, 1.70 mmol) were added. The reaction mixture was allowed to stir at room temperature for 24 h. The precipitate from the reaction mixture was vacuum filtered, washed with cold absolute EtOH (3 × 30 ml), and vacuum dried to give **II** as a dark-red solid (0.251 g, 75%). Red prisms suitable for single-crystal X-ray diffraction were obtained from diethyl ether solution by slow evaporation.

**3-diphenyl-6-mesitylfulvene (III).** To a vigorously stirred solution of 1,3-di­phenyl­cyclo­penta­diene (1.434 g, 6.57 mmol) in absolute EtOH (50 ml), mesityl­aldehyde (1.173 g, 7.91 mmol) and pyrrolidine (0.789 g, 11.09 mmol) were added. The reaction mixture was allowed to stir at reflux for 24 h. The reaction mixture was cooled to 278 K and the resulting precipitate was vacuum filtered, washed with cold absolute EtOH (3 × 30 ml), and vacuum dried to give **III** as a red–orange solid (1.85 g, 81%). Irregular red crystals suitable for single-crystal X-ray diffraction were obtained from pentane solution by slow evaporation.

**1,3-diphenyl-6-(2,3,4,5,6-penta­methyl­phen­yl)fulvene (IV).** To a vigorously stirred solution of 1,3-di­phenyl­cyclo­penta­diene (2.1 g, 9.62 mmol) in absolute EtOH (50 ml), 2,3,4,5,6-penta­methyl­benzaldehyde (2.04 g, 11.55 mmol) and pyrrolidine (1.09 g, 15.40 mmol) were added. The reaction mixture was allowed to stir at room temperature for 24 h. The precipitate from the reaction mixture was vacuum filtered, washed with cold absolute EtOH (3 × 30 ml), and vacuum dried to give **IV** as an orange solid (2.93 g, 82%). Orange needles suitable for single-crystal X-ray diffraction were obtained from ethyl acetate solution by slow evaporation.

## Refinement   

Crystal data, data collection and structure refinement details are summarized in Table 6 ▸. H atoms were positioned geometrically and refined as riding with C—H = 0.93–0.96 Å and *U*_iso_(H) = 1.2–1.5*U*_eq_(C). The absolute structure of **III** was indeterminate in the present refinement. Compound **III** was refined as an inversion twin.

## Supplementary Material

Crystal structure: contains datablock(s) I, II, III, IV. DOI: 10.1107/S205698901900700X/hb7824sup1.cif

Structure factors: contains datablock(s) I. DOI: 10.1107/S205698901900700X/hb7824Isup2.hkl

Structure factors: contains datablock(s) II. DOI: 10.1107/S205698901900700X/hb7824IIsup3.hkl

Structure factors: contains datablock(s) III. DOI: 10.1107/S205698901900700X/hb7824IIIsup4.hkl

Structure factors: contains datablock(s) IV. DOI: 10.1107/S205698901900700X/hb7824IVsup5.hkl

Supporting information file. DOI: 10.1107/S205698901900700X/hb7824Isup6.cml

Supporting information file. DOI: 10.1107/S205698901900700X/hb7824IIsup7.cml

Supporting information file. DOI: 10.1107/S205698901900700X/hb7824IIIsup8.cml

Supporting information file. DOI: 10.1107/S205698901900700X/hb7824IVsup9.cml

CCDC references: 1916092, 1916091, 1916090, 1916089

Additional supporting information:  crystallographic information; 3D view; checkCIF report

## Figures and Tables

**Figure 2:**
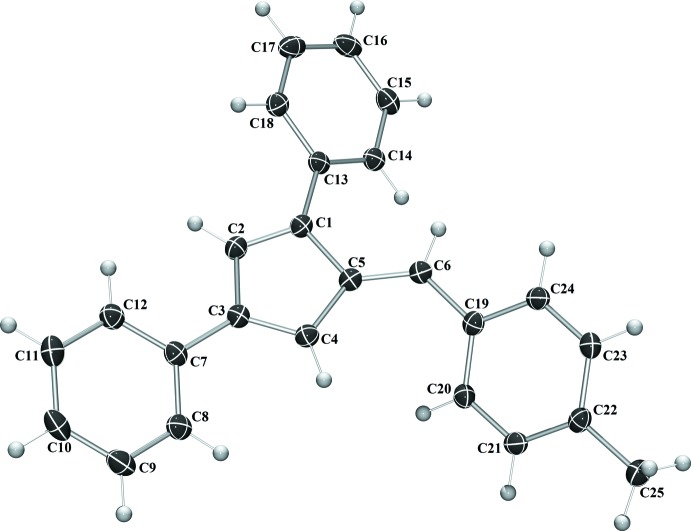
The mol­ecular structure of **II**. Displacement ellipsoids are shown at the 50% probability level.

**Figure 3:**
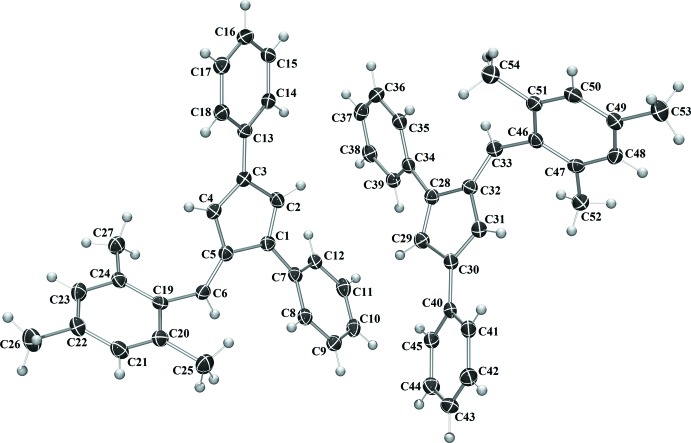
The mol­ecular structure of **III**. Displacement ellipsoids are shown at the 50% probability level.

**Figure 4:**
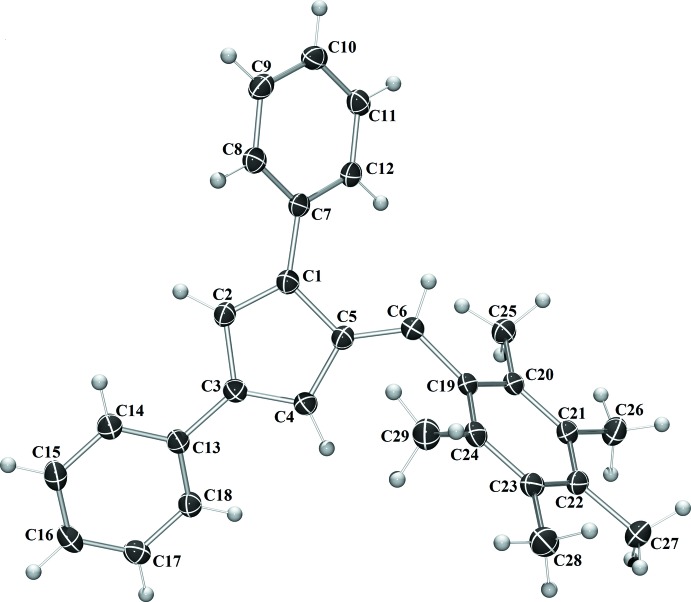
The mol­ecular structure of **IV**. Displacement ellipsoids are shown at the 50% probability level.

**Table 1:** Fulvene-phenyl torsion angles (°)

	**I**	**II**	**III**	**IV**
Fulvene*^*a*^*-(1-phen­yl*^*b*^*)	32.08 (7)	31.83 (5)	21.33 (13)	41.65 (7)
Fulvene*^*a*^*-(3-phen­yl*^*b*^*)	19.50 (6)	20.92 (5)	38.02 (13)	25.17 (7)
Fulvene*^*a*^*-(6-phen­yl*^*b*^*)	31.99 (6)	35.13 (5)	57.22 (14)	64.15 (7)

Notes: (*a*) plane defined by atoms C1–C5; (*a*) plane defined by the atoms of the specific phenyl ring substituent.

**Table 2:** Hydrogen-bond geometry (Å, °) for (I) *Cg*1 and *Cg*2 are the centroids of the C13–C18 and C19–C24 rings, respectively.

*D*—H⋯*A*	*D*—H	H⋯*A*	*D*⋯*A*	*D*—H⋯*A*
C10—H10⋯*Cg*1^i^	0.93	2.83	3.539 (2)	134
C16—H16⋯*Cg*2^ii^	0.93	2.86	3.589 (2)	136
C23—H23⋯*Cg*2^iii^	0.93	2.90	3.547 (2)	128

Symmetry codes: (i) 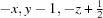; (ii) 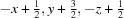; (iii) 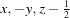.

**Table 3:** Hydrogen-bond geometry (Å, °) for (II) *Cg*3 and *Cg*4 are the centroids of the C7–C12 and C19–C24 rings, respectively.

*D*—H⋯*A*	*D*—H	H⋯*A*	*D*⋯*A*	*D*—H⋯*A*
C12—H12⋯*Cg*3^i^	0.95	2.95	3.6560 (4)	132
C17—H17⋯*Cg*3^ii^	0.95	2.83	3.4837 (4)	127
C25—H25*A*⋯*Cg*4^iii^	0.98	2.98	3.8277 (4)	145

Symmetry codes: (i) 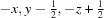; (ii) 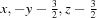; (iii) 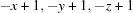.

**Table 4:** Hydrogen-bond geometry (Å, °) for (III) *Cg*5, *Cg*6, *Cg*7, *Cg*8, and *Cg*9 are the centroids of the C40–C45, C7–C12, C13–C18, C28–C32, and C34–C39 rings, respectively.

*D*—H⋯*A*	*D*—H	H⋯*A*	*D*⋯*A*	*D*—H⋯*A*
C10—H10⋯*Cg*5^i^	0.93	2.81	3.5059 (7)	132
C25—H25*C*⋯*Cg*6^ii^	0.96	2.81	3.7115 (8)	158
C37—H37⋯*Cg*7^iii^	0.93	2.71	3.5129 (8)	145
C39—H39⋯*Cg*8^ii^	0.93	2.97	3.5817 (8)	125
C53—H53*A*⋯*Cg*7^iv^	0.96	2.94	3.6503 (8)	132
C54—H54*C*⋯*Cg*9^v^	0.96	2.88	3.7983 (8)	160

Symmetry codes: (i) 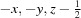; (ii) 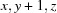; (iii) 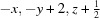; (iv) 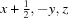; (v) 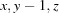.

**Table 5:** Hydrogen-bond geometry (Å, °) for (IV) *Cg*10 is the centroid of the C13–C18 ring.

*D*—H⋯*A*	*D*—H	H⋯*A*	*D*⋯*A*	*D*—H⋯*A*
C10—H10⋯*Cg*10^i^	0.95	2.70	3.4521 (9)	136

Symmetry code: (i) 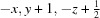.

**Table 6:** Experimental details

	(I)	(II)	(III)	(IV)
Crystal data				
Chemical formula	C_25_H_20_	C_25_H_20_	C_27_H_24_	C_29_H_28_
*M*_r_	320.41	320.41	348.46	376.51
Crystal system, space group	Monoclinic, *C*2/*c*	Monoclinic, *P*2_1_/*c*	Orthorhombic, *P* *c* *a*2_1_	Monoclinic, *C*2/*c*
Temperature (K)	100	100	100	100
*a*, *b*, *c* (Å)	29.230 (17), 5.800 (3), 22.071 (12)	19.208 (2), 5.8774 (7), 16.1884 (18)	30.031 (6), 5.6147 (12), 23.494 (5)	30.987 (7), 5.8273 (14), 23.557 (6)
α, β, γ (°)	90, 107.248 (17), 90	90, 107.710 (1), 90	90, 90, 90	90, 96.192 (3), 90
*V* (Å^3^)	3573 (3)	1740.9 (3)	3961.4 (14)	4228.9 (17)
*Z*	8	4	8	8
Radiation type	Mo *K*α	Mo *K*α	Mo *K*α	Mo *K*α
μ (mm^−1^)	0.07	0.07	0.07	0.07
Crystal size (mm)	0.25 × 0.21 × 0.18	0.43 × 0.27 × 0.06	0.26 × 0.11 × 0.10	0.44 × 0.11 × 0.1

Data collection				
Diffractometer	Bruker APEXII CCD	Bruker APEXII CCD	Bruker APEXII CCD	Bruker APEXII CCD
Absorption correction	Multi-scan *SADABS*	Multi-scan *SADABS*	Multi-scan *SADABS*	Multi-scan *SADABS*
*T*_min_, *T*_max_	0.832, 0.901	0.691, 0.745	0.678, 0.745	0.587, 0.745
No. of measured, independent and observed [*I* > 2σ(*I*)] reflections	31841, 3817, 3013	30237, 3473, 2972	38475, 6776, 5709	37965, 4394, 3213
*R*_int_	0.052	0.038	0.070	0.082
(sin θ/λ)_max_ (Å^−1^)	0.635	0.620	0.590	0.629

Refinement				
*R*[*F* ^2^ > 2σ(*F* ^2^)], *wR*(*F* ^2^), *S*	0.048, 0.122, 1.05	0.040, 0.100, 1.04	0.043, 0.108, 1.04	0.050, 0.137, 1.03
No. of reflections	3817	3473	6776	4394
No. of parameters	227	227	494	267
No. of restraints	0	0	1	0
H-atom treatment	H-atom parameters constrained	H-atom parameters constrained	H-atom parameters constrained	H-atom parameters constrained
Δρ_max_, Δρ_min_ (e Å^−3^)	0.33, −0.20	0.22, −0.19	0.17, −0.17	0.24, −0.22
Absolute structure	–	–	Refined as an inversion twin.	–
Absolute structure parameter	–	–	−6 (10)	–

Computer programs: *APEX3* and *SAINT* (Bruker, 2017 ▸), *SHELXT* (Sheldrick, 2015*a* ▸), *SHELXL* (Sheldrick, 2015*b* ▸), *ORTEP-3 for Windows* (Farrugia, 2012 ▸), *Mercury* (Macrae, *et al.*, 2008 ▸) and *publCIF* (Westrip, 2010 ▸).

## supplementary crystallographic information

### {3-[(3-Methylphenyl)methylidene]-4-phenylcyclopenta-1,4-dien-1-yl}benzene (I) Crystal data

**Table:**

C_25_H_20_	*F*(000) = 1360
*M**_r_* = 320.41	*D*_x_ = 1.191 Mg m^−^^3^
Monoclinic, *C*2/*c*	Mo *K*α radiation, λ = 0.71073 Å
*a* = 29.230 (17) Å	Cell parameters from 4339 reflections
*b* = 5.800 (3) Å	θ = 2.9–24.4°
*c* = 22.071 (12) Å	µ = 0.07 mm^−^^1^
β = 107.248 (17)°	*T* = 100 K
*V* = 3573 (3) Å^3^	Prism, red
*Z* = 8	0.25 × 0.21 × 0.18 mm

### {3-[(3-Methylphenyl)methylidene]-4-phenylcyclopenta-1,4-dien-1-yl}benzene (I) Data collection

**Table:**

Bruker APEXII CCD diffractometer	3013 reflections with *I* > 2σ(*I*)
φ and ω scans	*R*_int_ = 0.052
Absorption correction: multi-scan SADABS	θ_max_ = 26.8°, θ_min_ = 1.9°
*T*_min_ = 0.832, *T*_max_ = 0.901	*h* = −36→36
31841 measured reflections	*k* = −7→7
3817 independent reflections	*l* = −28→27

### {3-[(3-Methylphenyl)methylidene]-4-phenylcyclopenta-1,4-dien-1-yl}benzene (I) Refinement

**Table:**

Refinement on *F*^2^	0 restraints
Least-squares matrix: full	Hydrogen site location: inferred from neighbouring sites
*R*[*F*^2^ > 2σ(*F*^2^)] = 0.048	H-atom parameters constrained
*wR*(*F*^2^) = 0.122	*w* = 1/[σ^2^(*F*_o_^2^) + (0.0482*P*)^2^ + 3.8961*P*] where *P* = (*F*_o_^2^ + 2*F*_c_^2^)/3
*S* = 1.05	(Δ/σ)_max_ < 0.001
3817 reflections	Δρ_max_ = 0.33 e Å^−^^3^
227 parameters	Δρ_min_ = −0.19 e Å^−^^3^

### {3-[(3-Methylphenyl)methylidene]-4-phenylcyclopenta-1,4-dien-1-yl}benzene (I) Special details

**Table:**

Geometry. All esds (except the esd in the dihedral angle between two l.s. planes) are estimated using the full covariance matrix. The cell esds are taken into account individually in the estimation of esds in distances, angles and torsion angles; correlations between esds in cell parameters are only used when they are defined by crystal symmetry. An approximate (isotropic) treatment of cell esds is used for estimating esds involving l.s. planes.

### {3-[(3-Methylphenyl)methylidene]-4-phenylcyclopenta-1,4-dien-1-yl}benzene (I) Fractional atomic coordinates and isotropic or equivalent isotropic displacement parameters (Å^2^)

**Table:**

	*x*	*y*	*z*	*U*_iso_*/*U*_eq_	
C1	0.57871 (5)	0.4490 (3)	0.23560 (7)	0.0219 (3)	
C2	0.57462 (5)	0.4576 (3)	0.29513 (7)	0.0229 (3)	
H2	0.554463	0.364773	0.310252	0.027*	
C3	0.60677 (5)	0.6356 (3)	0.33229 (7)	0.0222 (3)	
C4	0.62991 (5)	0.7360 (3)	0.29436 (7)	0.0235 (3)	
H4	0.651702	0.856706	0.305993	0.028*	
C5	0.61519 (5)	0.6246 (3)	0.23236 (7)	0.0221 (3)	
C6	0.63418 (5)	0.6503 (3)	0.18410 (7)	0.0238 (3)	
H6	0.623184	0.548206	0.150415	0.029*	
C7	0.55041 (5)	0.2988 (3)	0.18388 (7)	0.0218 (3)	
C8	0.53551 (5)	0.3688 (3)	0.12045 (7)	0.0255 (3)	
H8	0.544591	0.512625	0.109349	0.031*	
C9	0.50730 (6)	0.2255 (3)	0.07390 (8)	0.0303 (4)	
H9	0.498411	0.272695	0.031741	0.036*	
C10	0.49212 (6)	0.0122 (3)	0.08943 (8)	0.0306 (4)	
H10	0.472725	−0.081758	0.058066	0.037*	
C11	0.50628 (6)	−0.0585 (3)	0.15223 (8)	0.0275 (4)	
H11	0.496034	−0.199944	0.163231	0.033*	
C12	0.53569 (5)	0.0812 (3)	0.19877 (7)	0.0235 (3)	
H12	0.545815	0.029678	0.240537	0.028*	
C13	0.61345 (5)	0.6870 (3)	0.39949 (7)	0.0235 (3)	
C14	0.59996 (6)	0.5279 (3)	0.43855 (7)	0.0269 (4)	
H14	0.586124	0.388692	0.421668	0.032*	
C15	0.60698 (6)	0.5755 (3)	0.50228 (8)	0.0320 (4)	
H15	0.597703	0.468403	0.527717	0.038*	
C16	0.62776 (6)	0.7819 (3)	0.52829 (8)	0.0331 (4)	
H16	0.632614	0.813134	0.571065	0.040*	
C17	0.64122 (6)	0.9410 (3)	0.49002 (8)	0.0334 (4)	
H17	0.655280	1.079353	0.507240	0.040*	
C18	0.63390 (6)	0.8956 (3)	0.42650 (8)	0.0293 (4)	
H18	0.642674	1.004989	0.401210	0.035*	
C19	0.67002 (5)	0.8189 (3)	0.17789 (7)	0.0233 (3)	
C20	0.69945 (5)	0.7614 (3)	0.14047 (7)	0.0255 (3)	
H20	0.694917	0.620567	0.119385	0.031*	
C21	0.73507 (6)	0.9072 (3)	0.13377 (7)	0.0279 (4)	
C22	0.74101 (6)	1.1205 (3)	0.16461 (8)	0.0302 (4)	
H22	0.764784	1.220841	0.160703	0.036*	
C23	0.71154 (6)	1.1835 (3)	0.20115 (7)	0.0272 (4)	
H23	0.715732	1.325711	0.221509	0.033*	
C24	0.67616 (6)	1.0360 (3)	0.20731 (7)	0.0248 (3)	
H24	0.656224	1.080714	0.231134	0.030*	
C25	0.76705 (7)	0.8402 (4)	0.09398 (10)	0.0443 (5)	
H25A	0.759165	0.687097	0.077736	0.066*	
H25B	0.799917	0.844814	0.119636	0.066*	
H25C	0.762396	0.946296	0.059275	0.066*	

### {3-[(3-Methylphenyl)methylidene]-4-phenylcyclopenta-1,4-dien-1-yl}benzene (I) Atomic displacement parameters (Å^2^)

**Table:**

	*U*^11^	*U*^22^	*U*^33^	*U*^12^	*U*^13^	*U*^23^
C1	0.0181 (7)	0.0214 (8)	0.0268 (8)	0.0013 (6)	0.0074 (6)	0.0003 (6)
C2	0.0207 (7)	0.0242 (8)	0.0245 (8)	−0.0022 (6)	0.0080 (6)	−0.0003 (6)
C3	0.0182 (7)	0.0229 (8)	0.0250 (8)	0.0014 (6)	0.0055 (6)	0.0006 (6)
C4	0.0201 (7)	0.0236 (8)	0.0252 (8)	0.0000 (6)	0.0045 (6)	0.0001 (6)
C5	0.0197 (7)	0.0213 (8)	0.0255 (8)	0.0025 (6)	0.0072 (6)	0.0026 (6)
C6	0.0235 (8)	0.0237 (8)	0.0233 (7)	0.0017 (6)	0.0055 (6)	−0.0023 (6)
C7	0.0185 (7)	0.0231 (8)	0.0255 (8)	0.0025 (6)	0.0092 (6)	−0.0032 (6)
C8	0.0252 (8)	0.0258 (8)	0.0275 (8)	−0.0001 (7)	0.0108 (7)	−0.0005 (6)
C9	0.0294 (9)	0.0375 (10)	0.0241 (8)	0.0027 (7)	0.0083 (7)	−0.0033 (7)
C10	0.0263 (8)	0.0326 (9)	0.0322 (9)	−0.0029 (7)	0.0074 (7)	−0.0127 (7)
C11	0.0256 (8)	0.0225 (8)	0.0362 (9)	−0.0012 (7)	0.0120 (7)	−0.0056 (7)
C12	0.0215 (7)	0.0244 (8)	0.0261 (8)	0.0025 (6)	0.0093 (6)	−0.0013 (6)
C13	0.0199 (7)	0.0267 (8)	0.0227 (7)	0.0013 (6)	0.0046 (6)	−0.0009 (6)
C14	0.0304 (8)	0.0246 (8)	0.0251 (8)	−0.0017 (7)	0.0074 (7)	−0.0015 (6)
C15	0.0404 (10)	0.0328 (9)	0.0231 (8)	0.0005 (8)	0.0102 (7)	0.0035 (7)
C16	0.0404 (10)	0.0361 (10)	0.0200 (8)	0.0021 (8)	0.0045 (7)	−0.0016 (7)
C17	0.0369 (9)	0.0322 (9)	0.0271 (8)	−0.0048 (8)	0.0035 (7)	−0.0067 (7)
C18	0.0315 (9)	0.0278 (9)	0.0278 (8)	−0.0035 (7)	0.0076 (7)	0.0012 (7)
C19	0.0246 (8)	0.0245 (8)	0.0194 (7)	0.0008 (6)	0.0045 (6)	0.0022 (6)
C20	0.0264 (8)	0.0255 (8)	0.0242 (8)	0.0020 (7)	0.0068 (6)	0.0004 (6)
C21	0.0254 (8)	0.0344 (9)	0.0246 (8)	0.0021 (7)	0.0087 (6)	0.0037 (7)
C22	0.0242 (8)	0.0316 (9)	0.0324 (9)	−0.0064 (7)	0.0047 (7)	0.0068 (7)
C23	0.0274 (8)	0.0239 (8)	0.0271 (8)	0.0006 (7)	0.0029 (6)	0.0000 (6)
C24	0.0279 (8)	0.0267 (8)	0.0210 (7)	0.0012 (7)	0.0090 (6)	0.0016 (6)
C25	0.0418 (11)	0.0486 (12)	0.0519 (12)	−0.0053 (9)	0.0283 (9)	−0.0048 (10)

### {3-[(3-Methylphenyl)methylidene]-4-phenylcyclopenta-1,4-dien-1-yl}benzene (I) Geometric parameters (Å, º)

**Table:**

C1—C2	1.355 (2)	C13—C18	1.402 (2)
C1—C5	1.492 (2)	C14—H14	0.9300
C1—C7	1.478 (2)	C14—C15	1.388 (2)
C2—H2	0.9300	C15—H15	0.9300
C2—C3	1.471 (2)	C15—C16	1.387 (3)
C3—C4	1.354 (2)	C16—H16	0.9300
C3—C13	1.468 (2)	C16—C17	1.384 (3)
C4—H4	0.9300	C17—H17	0.9300
C4—C5	1.458 (2)	C17—C18	1.379 (2)
C5—C6	1.347 (2)	C18—H18	0.9300
C6—H6	0.9300	C19—C20	1.398 (2)
C6—C19	1.469 (2)	C19—C24	1.403 (2)
C7—C8	1.397 (2)	C20—H20	0.9300
C7—C12	1.404 (2)	C20—C21	1.383 (2)
C8—H8	0.9300	C21—C22	1.398 (2)
C8—C9	1.387 (2)	C21—C25	1.511 (2)
C9—H9	0.9300	C22—H22	0.9300
C9—C10	1.391 (3)	C22—C23	1.392 (2)
C10—H10	0.9300	C23—H23	0.9300
C10—C11	1.386 (2)	C23—C24	1.380 (2)
C11—H11	0.9300	C24—H24	0.9300
C11—C12	1.388 (2)	C25—H25A	0.9600
C12—H12	0.9300	C25—H25B	0.9600
C13—C14	1.397 (2)	C25—H25C	0.9600
			
C2—C1—C5	106.92 (13)	C13—C14—H14	119.7
C2—C1—C7	125.48 (14)	C15—C14—C13	120.67 (16)
C7—C1—C5	127.55 (13)	C15—C14—H14	119.7
C1—C2—H2	125.0	C14—C15—H15	119.8
C1—C2—C3	109.94 (14)	C16—C15—C14	120.40 (16)
C3—C2—H2	125.0	C16—C15—H15	119.8
C4—C3—C2	108.10 (14)	C15—C16—H16	120.3
C4—C3—C13	126.58 (14)	C17—C16—C15	119.43 (16)
C13—C3—C2	125.28 (14)	C17—C16—H16	120.3
C3—C4—H4	125.4	C16—C17—H17	119.8
C3—C4—C5	109.10 (14)	C18—C17—C16	120.43 (16)
C5—C4—H4	125.4	C18—C17—H17	119.8
C4—C5—C1	105.89 (13)	C13—C18—H18	119.5
C6—C5—C1	125.53 (14)	C17—C18—C13	121.01 (16)
C6—C5—C4	128.10 (15)	C17—C18—H18	119.5
C5—C6—H6	115.9	C20—C19—C6	118.51 (14)
C5—C6—C19	128.29 (15)	C20—C19—C24	118.07 (14)
C19—C6—H6	115.9	C24—C19—C6	123.42 (14)
C8—C7—C1	122.65 (14)	C19—C20—H20	118.9
C8—C7—C12	118.00 (14)	C21—C20—C19	122.15 (15)
C12—C7—C1	119.28 (14)	C21—C20—H20	118.9
C7—C8—H8	119.7	C20—C21—C22	118.53 (15)
C9—C8—C7	120.56 (15)	C20—C21—C25	121.35 (16)
C9—C8—H8	119.7	C22—C21—C25	120.11 (16)
C8—C9—H9	119.6	C21—C22—H22	119.8
C8—C9—C10	120.89 (16)	C23—C22—C21	120.38 (15)
C10—C9—H9	119.6	C23—C22—H22	119.8
C9—C10—H10	120.4	C22—C23—H23	119.8
C11—C10—C9	119.15 (15)	C24—C23—C22	120.34 (16)
C11—C10—H10	120.4	C24—C23—H23	119.8
C10—C11—H11	119.9	C19—C24—H24	119.8
C10—C11—C12	120.22 (16)	C23—C24—C19	120.48 (15)
C12—C11—H11	119.9	C23—C24—H24	119.8
C7—C12—H12	119.4	C21—C25—H25A	109.5
C11—C12—C7	121.14 (15)	C21—C25—H25B	109.5
C11—C12—H12	119.4	C21—C25—H25C	109.5
C14—C13—C3	120.79 (14)	H25A—C25—H25B	109.5
C14—C13—C18	118.05 (15)	H25A—C25—H25C	109.5
C18—C13—C3	121.16 (14)	H25B—C25—H25C	109.5

### {3-[(3-Methylphenyl)methylidene]-4-phenylcyclopenta-1,4-dien-1-yl}benzene (I) Hydrogen-bond geometry (Å, º)

*Cg*1 and *Cg*2 are the centroids of the C13–C18 and C19–C24 rings, respectively.

**Table:**

*D*—H···*A*	*D*—H	H···*A*	*D*···*A*	*D*—H···*A*
C10—H10···*Cg*1^i^	0.93	2.83	3.539 (2)	134
C16—H16···*Cg*2^ii^	0.93	2.86	3.589 (2)	136
C23—H23···*Cg*2^iii^	0.93	2.90	3.547 (2)	128

Symmetry codes: (i) −*x*, *y*−1, −*z*+1/2; (ii) −*x*+1/2, *y*+3/2, −*z*+1/2; (iii) *x*, −*y*, *z*−1/2.

### {3-[(4-Methylphenyl)methylidene]-4-phenylcyclopenta-1,4-dien-1-yl}benzene (II) Crystal data

**Table:**

C_25_H_20_	*F*(000) = 680
*M**_r_* = 320.41	*D*_x_ = 1.222 Mg m^−^^3^
Monoclinic, *P*2_1_/*c*	Mo *K*α radiation, λ = 0.71073 Å
*a* = 19.208 (2) Å	Cell parameters from 6861 reflections
*b* = 5.8774 (7) Å	θ = 2.2–26.1°
*c* = 16.1884 (18) Å	µ = 0.07 mm^−^^1^
β = 107.710 (1)°	*T* = 100 K
*V* = 1740.9 (3) Å^3^	Rect. Prism, red
*Z* = 4	0.43 × 0.27 × 0.06 mm

### {3-[(4-Methylphenyl)methylidene]-4-phenylcyclopenta-1,4-dien-1-yl}benzene (II) Data collection

**Table:**

Bruker APEXII CCD diffractometer	2972 reflections with *I* > 2σ(*I*)
Radiation source: fine focus sealed tube	*R*_int_ = 0.038
φ and ω scans	θ_max_ = 26.2°, θ_min_ = 2.2°
Absorption correction: multi-scan SADABS	*h* = −23→23
*T*_min_ = 0.691, *T*_max_ = 0.745	*k* = −7→7
30237 measured reflections	*l* = −20→20
3473 independent reflections	

### {3-[(4-Methylphenyl)methylidene]-4-phenylcyclopenta-1,4-dien-1-yl}benzene (II) Refinement

**Table:**

Refinement on *F*^2^	Primary atom site location: dual
Least-squares matrix: full	Hydrogen site location: inferred from neighbouring sites
*R*[*F*^2^ > 2σ(*F*^2^)] = 0.040	H-atom parameters constrained
*wR*(*F*^2^) = 0.100	*w* = 1/[σ^2^(*F*_o_^2^) + (0.0394*P*)^2^ + 0.8955*P*] where *P* = (*F*_o_^2^ + 2*F*_c_^2^)/3
*S* = 1.04	(Δ/σ)_max_ < 0.001
3473 reflections	Δρ_max_ = 0.22 e Å^−^^3^
227 parameters	Δρ_min_ = −0.19 e Å^−^^3^
0 restraints	

### {3-[(4-Methylphenyl)methylidene]-4-phenylcyclopenta-1,4-dien-1-yl}benzene (II) Special details

**Table:**

Geometry. All esds (except the esd in the dihedral angle between two l.s. planes) are estimated using the full covariance matrix. The cell esds are taken into account individually in the estimation of esds in distances, angles and torsion angles; correlations between esds in cell parameters are only used when they are defined by crystal symmetry. An approximate (isotropic) treatment of cell esds is used for estimating esds involving l.s. planes.

### {3-[(4-Methylphenyl)methylidene]-4-phenylcyclopenta-1,4-dien-1-yl}benzene (II) Fractional atomic coordinates and isotropic or equivalent isotropic displacement parameters (Å^2^)

**Table:**

	*x*	*y*	*z*	*U*_iso_*/*U*_eq_	
C1	0.21805 (7)	0.4854 (2)	0.67244 (8)	0.0199 (3)	
C19	0.39564 (7)	0.1392 (2)	0.78528 (8)	0.0196 (3)	
C3	0.16408 (7)	0.3062 (2)	0.76451 (8)	0.0199 (3)	
C13	0.23043 (6)	0.6328 (2)	0.60483 (8)	0.0199 (3)	
C24	0.46911 (7)	0.2083 (2)	0.81558 (8)	0.0205 (3)	
H24	0.482874	0.351246	0.797988	0.025*	
C6	0.34162 (7)	0.2980 (2)	0.73220 (8)	0.0199 (3)	
H6	0.358882	0.400502	0.697460	0.024*	
C21	0.43165 (7)	−0.2155 (2)	0.86275 (8)	0.0217 (3)	
H21	0.418584	−0.361899	0.878004	0.026*	
C22	0.50403 (7)	−0.1435 (2)	0.89566 (8)	0.0209 (3)	
C23	0.52175 (7)	0.0712 (2)	0.87068 (8)	0.0212 (3)	
H23	0.570728	0.123926	0.891873	0.025*	
C4	0.23106 (7)	0.2078 (2)	0.78063 (8)	0.0205 (3)	
H4	0.249483	0.087659	0.820575	0.025*	
C14	0.27283 (7)	0.5647 (2)	0.55254 (8)	0.0218 (3)	
H14	0.294824	0.418298	0.560510	0.026*	
C7	0.11136 (7)	0.2699 (2)	0.81300 (8)	0.0205 (3)	
C5	0.26996 (7)	0.3166 (2)	0.72660 (8)	0.0195 (3)	
C20	0.37819 (7)	−0.0787 (2)	0.80827 (8)	0.0206 (3)	
H20	0.329440	−0.133081	0.786414	0.025*	
C2	0.15635 (7)	0.4766 (2)	0.69629 (8)	0.0217 (3)	
H2	0.114171	0.567633	0.672247	0.026*	
C18	0.19736 (7)	0.8485 (2)	0.58962 (8)	0.0229 (3)	
H18	0.168610	0.899257	0.624501	0.027*	
C8	0.11440 (7)	0.0724 (2)	0.86246 (8)	0.0240 (3)	
H8	0.146980	−0.046782	0.859555	0.029*	
C12	0.06039 (7)	0.4379 (2)	0.81513 (8)	0.0235 (3)	
H12	0.055986	0.569546	0.779883	0.028*	
C17	0.20606 (7)	0.9880 (2)	0.52453 (9)	0.0277 (3)	
H17	0.181975	1.131132	0.513845	0.033*	
C15	0.28318 (7)	0.7086 (2)	0.48909 (8)	0.0256 (3)	
H15	0.313325	0.661580	0.455334	0.031*	
C9	0.07027 (7)	0.0496 (3)	0.91556 (9)	0.0284 (3)	
H9	0.073164	−0.084411	0.949270	0.034*	
C11	0.01606 (7)	0.4138 (3)	0.86843 (9)	0.0272 (3)	
H11	−0.018208	0.529317	0.869678	0.033*	
C16	0.24982 (7)	0.9197 (2)	0.47484 (9)	0.0285 (3)	
H16	0.256832	1.017343	0.431291	0.034*	
C10	0.02185 (7)	0.2213 (3)	0.91982 (9)	0.0289 (3)	
H10	−0.007197	0.207094	0.957700	0.035*	
C25	0.56192 (7)	−0.2930 (2)	0.95447 (9)	0.0277 (3)	
H25A	0.582960	−0.216495	1.010312	0.042*	
H25B	0.600443	−0.322080	0.927669	0.042*	
H25C	0.539995	−0.437632	0.963685	0.042*	

### {3-[(4-Methylphenyl)methylidene]-4-phenylcyclopenta-1,4-dien-1-yl}benzene (II) Atomic displacement parameters (Å^2^)

**Table:**

	*U*^11^	*U*^22^	*U*^33^	*U*^12^	*U*^13^	*U*^23^
C1	0.0197 (6)	0.0202 (6)	0.0184 (6)	0.0004 (5)	0.0035 (5)	−0.0010 (5)
C19	0.0201 (6)	0.0221 (6)	0.0176 (6)	0.0027 (5)	0.0074 (5)	−0.0014 (5)
C3	0.0196 (6)	0.0205 (6)	0.0185 (6)	−0.0006 (5)	0.0043 (5)	−0.0015 (5)
C13	0.0158 (6)	0.0224 (6)	0.0188 (6)	−0.0023 (5)	0.0013 (5)	0.0012 (5)
C24	0.0224 (6)	0.0192 (6)	0.0219 (6)	0.0011 (5)	0.0096 (5)	0.0003 (5)
C6	0.0219 (6)	0.0201 (6)	0.0182 (6)	0.0004 (5)	0.0067 (5)	−0.0004 (5)
C21	0.0240 (6)	0.0196 (6)	0.0241 (6)	0.0017 (5)	0.0113 (5)	0.0014 (5)
C22	0.0220 (6)	0.0231 (6)	0.0190 (6)	0.0053 (5)	0.0084 (5)	0.0009 (5)
C23	0.0177 (6)	0.0242 (7)	0.0221 (6)	0.0007 (5)	0.0065 (5)	−0.0024 (5)
C4	0.0223 (6)	0.0200 (6)	0.0191 (6)	0.0009 (5)	0.0060 (5)	0.0006 (5)
C14	0.0190 (6)	0.0238 (7)	0.0207 (6)	−0.0010 (5)	0.0033 (5)	0.0011 (5)
C7	0.0174 (6)	0.0245 (7)	0.0180 (6)	−0.0034 (5)	0.0030 (5)	−0.0011 (5)
C5	0.0211 (6)	0.0193 (6)	0.0176 (6)	0.0006 (5)	0.0051 (5)	−0.0011 (5)
C20	0.0185 (6)	0.0215 (6)	0.0225 (6)	0.0005 (5)	0.0073 (5)	−0.0015 (5)
C2	0.0191 (6)	0.0228 (7)	0.0220 (6)	0.0023 (5)	0.0046 (5)	0.0022 (5)
C18	0.0195 (6)	0.0230 (7)	0.0242 (6)	−0.0005 (5)	0.0038 (5)	−0.0008 (5)
C8	0.0194 (6)	0.0262 (7)	0.0240 (6)	−0.0027 (5)	0.0028 (5)	0.0017 (5)
C12	0.0210 (6)	0.0252 (7)	0.0238 (6)	−0.0014 (5)	0.0058 (5)	0.0008 (5)
C17	0.0253 (7)	0.0224 (7)	0.0294 (7)	−0.0016 (6)	−0.0007 (6)	0.0049 (6)
C15	0.0214 (6)	0.0348 (8)	0.0196 (6)	−0.0048 (6)	0.0046 (5)	0.0012 (6)
C9	0.0243 (7)	0.0337 (8)	0.0253 (7)	−0.0066 (6)	0.0049 (5)	0.0066 (6)
C11	0.0201 (6)	0.0338 (8)	0.0281 (7)	−0.0009 (6)	0.0080 (5)	−0.0042 (6)
C16	0.0278 (7)	0.0305 (8)	0.0232 (7)	−0.0074 (6)	0.0021 (5)	0.0088 (6)
C10	0.0211 (7)	0.0419 (8)	0.0249 (7)	−0.0083 (6)	0.0091 (5)	0.0000 (6)
C25	0.0228 (7)	0.0290 (7)	0.0305 (7)	0.0040 (6)	0.0067 (6)	0.0063 (6)

### {3-[(4-Methylphenyl)methylidene]-4-phenylcyclopenta-1,4-dien-1-yl}benzene (II) Geometric parameters (Å, º)

**Table:**

C1—C13	1.4707 (17)	C14—C15	1.3906 (18)
C1—C5	1.4883 (17)	C7—C8	1.4012 (18)
C1—C2	1.3547 (18)	C7—C12	1.3981 (19)
C19—C24	1.4057 (17)	C20—H20	0.9500
C19—C6	1.4642 (17)	C2—H2	0.9500
C19—C20	1.4023 (18)	C18—H18	0.9500
C3—C4	1.3616 (17)	C18—C17	1.3850 (19)
C3—C7	1.4733 (17)	C8—H8	0.9500
C3—C2	1.4641 (17)	C8—C9	1.3850 (19)
C13—C14	1.3997 (18)	C12—H12	0.9500
C13—C18	1.4056 (18)	C12—C11	1.3916 (18)
C24—H24	0.9500	C17—H17	0.9500
C24—C23	1.3852 (18)	C17—C16	1.388 (2)
C6—H6	0.9500	C15—H15	0.9500
C6—C5	1.3561 (17)	C15—C16	1.383 (2)
C21—H21	0.9500	C9—H9	0.9500
C21—C22	1.3946 (18)	C9—C10	1.388 (2)
C21—C20	1.3883 (18)	C11—H11	0.9500
C22—C23	1.3983 (19)	C11—C10	1.389 (2)
C22—C25	1.5068 (17)	C16—H16	0.9500
C23—H23	0.9500	C10—H10	0.9500
C4—H4	0.9500	C25—H25A	0.9800
C4—C5	1.4596 (17)	C25—H25B	0.9800
C14—H14	0.9500	C25—H25C	0.9800
			
C13—C1—C5	126.87 (11)	C19—C20—H20	119.8
C2—C1—C13	126.04 (12)	C21—C20—C19	120.46 (12)
C2—C1—C5	107.08 (11)	C21—C20—H20	119.8
C24—C19—C6	118.52 (12)	C1—C2—C3	110.16 (11)
C20—C19—C24	117.84 (11)	C1—C2—H2	124.9
C20—C19—C6	123.64 (11)	C3—C2—H2	124.9
C4—C3—C7	126.77 (12)	C13—C18—H18	119.5
C4—C3—C2	107.96 (11)	C17—C18—C13	120.97 (13)
C2—C3—C7	124.93 (11)	C17—C18—H18	119.5
C14—C13—C1	122.49 (12)	C7—C8—H8	119.7
C14—C13—C18	117.84 (12)	C9—C8—C7	120.53 (13)
C18—C13—C1	119.65 (12)	C9—C8—H8	119.7
C19—C24—H24	119.5	C7—C12—H12	119.7
C23—C24—C19	121.01 (12)	C11—C12—C7	120.58 (13)
C23—C24—H24	119.5	C11—C12—H12	119.7
C19—C6—H6	116.0	C18—C17—H17	119.9
C5—C6—C19	127.90 (12)	C18—C17—C16	120.26 (13)
C5—C6—H6	116.0	C16—C17—H17	119.9
C22—C21—H21	119.1	C14—C15—H15	119.8
C20—C21—H21	119.1	C16—C15—C14	120.36 (13)
C20—C21—C22	121.73 (12)	C16—C15—H15	119.8
C21—C22—C23	117.72 (12)	C8—C9—H9	119.8
C21—C22—C25	121.52 (12)	C8—C9—C10	120.40 (13)
C23—C22—C25	120.74 (12)	C10—C9—H9	119.8
C24—C23—C22	121.14 (12)	C12—C11—H11	120.0
C24—C23—H23	119.4	C10—C11—C12	120.09 (13)
C22—C23—H23	119.4	C10—C11—H11	120.0
C3—C4—H4	125.5	C17—C16—H16	120.2
C3—C4—C5	108.96 (11)	C15—C16—C17	119.64 (13)
C5—C4—H4	125.5	C15—C16—H16	120.2
C13—C14—H14	119.6	C9—C10—C11	119.70 (13)
C15—C14—C13	120.87 (13)	C9—C10—H10	120.2
C15—C14—H14	119.6	C11—C10—H10	120.2
C8—C7—C3	120.58 (12)	C22—C25—H25A	109.5
C12—C7—C3	120.70 (12)	C22—C25—H25B	109.5
C12—C7—C8	118.59 (12)	C22—C25—H25C	109.5
C6—C5—C1	125.28 (12)	H25A—C25—H25B	109.5
C6—C5—C4	128.23 (12)	H25A—C25—H25C	109.5
C4—C5—C1	105.78 (10)	H25B—C25—H25C	109.5

### {3-[(4-Methylphenyl)methylidene]-4-phenylcyclopenta-1,4-dien-1-yl}benzene (II) Hydrogen-bond geometry (Å, º)

*Cg*3 and *Cg*4 are the centroids of the C7–C12 and C19–C24 rings, respectively.

**Table:**

*D*—H···*A*	*D*—H	H···*A*	*D*···*A*	*D*—H···*A*
C12—H12···*Cg*3^i^	0.95	2.95	3.6560 (4)	132
C17—H17···*Cg*3^ii^	0.95	2.83	3.4837 (4)	127
C25—H25*A*···*Cg*4^iii^	0.98	2.98	3.8277 (4)	145

Symmetry codes: (i) −*x*, *y*−1/2, −*z*+1/2; (ii) *x*, −*y*−3/2, *z*−3/2; (iii) −*x*+1, −*y*+1, −*z*+1.

### {3-[(2,4,6-Trimethylphenyl)methylidene]-4-phenylcyclopenta-1,4-dien-1-yl}benzene (III) Crystal data

**Table:**

C_27_H_24_	*D*_x_ = 1.169 Mg m^−^^3^
*M**_r_* = 348.46	Mo *K*α radiation, λ = 0.71073 Å
Orthorhombic, *P**c**a*2_1_	Cell parameters from 3728 reflections
*a* = 30.031 (6) Å	θ = 2.2–22.3°
*b* = 5.6147 (12) Å	µ = 0.07 mm^−^^1^
*c* = 23.494 (5) Å	*T* = 100 K
*V* = 3961.4 (14) Å^3^	Irregular, red
*Z* = 8	0.26 × 0.11 × 0.10 mm
*F*(000) = 1488	

### {3-[(2,4,6-Trimethylphenyl)methylidene]-4-phenylcyclopenta-1,4-dien-1-yl}benzene (III) Data collection

**Table:**

Bruker APEXII CCD diffractometer	5709 reflections with *I* > 2σ(*I*)
φ and ω scans	*R*_int_ = 0.070
Absorption correction: multi-scan SADABS	θ_max_ = 24.8°, θ_min_ = 2.2°
*T*_min_ = 0.678, *T*_max_ = 0.745	*h* = −35→35
38475 measured reflections	*k* = −6→6
6776 independent reflections	*l* = −27→27

### {3-[(2,4,6-Trimethylphenyl)methylidene]-4-phenylcyclopenta-1,4-dien-1-yl}benzene (III) Refinement

**Table:**

Refinement on *F*^2^	Hydrogen site location: inferred from neighbouring sites
Least-squares matrix: full	H-atom parameters constrained
*R*[*F*^2^ > 2σ(*F*^2^)] = 0.043	*w* = 1/[σ^2^(*F*_o_^2^) + (0.054*P*)^2^ + 0.3457*P*] where *P* = (*F*_o_^2^ + 2*F*_c_^2^)/3
*wR*(*F*^2^) = 0.108	(Δ/σ)_max_ = 0.001
*S* = 1.03	Δρ_max_ = 0.17 e Å^−^^3^
6776 reflections	Δρ_min_ = −0.17 e Å^−^^3^
494 parameters	Absolute structure: Refined as an inversion twin.
1 restraint	Absolute structure parameter: −6 (10)
Primary atom site location: dual	

### {3-[(2,4,6-Trimethylphenyl)methylidene]-4-phenylcyclopenta-1,4-dien-1-yl}benzene (III) Special details

**Table:**

Geometry. All esds (except the esd in the dihedral angle between two l.s. planes) are estimated using the full covariance matrix. The cell esds are taken into account individually in the estimation of esds in distances, angles and torsion angles; correlations between esds in cell parameters are only used when they are defined by crystal symmetry. An approximate (isotropic) treatment of cell esds is used for estimating esds involving l.s. planes.
Refinement. Refined as a two-component inversion twin

### {3-[(2,4,6-Trimethylphenyl)methylidene]-4-phenylcyclopenta-1,4-dien-1-yl}benzene (III) Fractional atomic coordinates and isotropic or equivalent isotropic displacement parameters (Å^2^)

**Table:**

	*x*	*y*	*z*	*U*_iso_*/*U*_eq_	
C1	0.35712 (11)	0.1134 (6)	0.54077 (15)	0.0224 (8)	
C2	0.36382 (11)	0.1309 (6)	0.48394 (15)	0.0238 (8)	
H2	0.384904	0.045102	0.463428	0.029*	
C3	0.33274 (11)	0.3060 (6)	0.45947 (15)	0.0219 (8)	
C4	0.30771 (12)	0.3951 (6)	0.50228 (15)	0.0230 (8)	
H4	0.286286	0.513761	0.498702	0.028*	
C5	0.31988 (11)	0.2742 (6)	0.55511 (15)	0.0227 (8)	
C6	0.29829 (11)	0.2883 (6)	0.60543 (15)	0.0239 (8)	
H6	0.308796	0.193941	0.635065	0.029*	
C7	0.38365 (11)	−0.0333 (6)	0.58024 (14)	0.0225 (8)	
C8	0.39541 (11)	0.0453 (6)	0.63499 (15)	0.0245 (8)	
H8	0.385132	0.191708	0.648020	0.029*	
C9	0.42211 (12)	−0.0930 (6)	0.66963 (16)	0.0274 (8)	
H9	0.429090	−0.040616	0.706110	0.033*	
C10	0.43847 (12)	−0.3077 (7)	0.65055 (17)	0.0283 (9)	
H10	0.456900	−0.398471	0.673806	0.034*	
C11	0.42743 (12)	−0.3879 (6)	0.59677 (16)	0.0265 (9)	
H11	0.438662	−0.532334	0.583768	0.032*	
C12	0.39980 (11)	−0.2547 (6)	0.56223 (15)	0.0227 (8)	
H12	0.391827	−0.312675	0.526587	0.027*	
C13	0.33002 (11)	0.3610 (6)	0.39856 (15)	0.0216 (8)	
C14	0.34639 (11)	0.1972 (6)	0.35861 (14)	0.0243 (8)	
H14	0.359649	0.057076	0.371174	0.029*	
C15	0.34310 (12)	0.2409 (6)	0.30085 (16)	0.0284 (8)	
H15	0.354276	0.130280	0.275078	0.034*	
C16	0.32342 (12)	0.4469 (7)	0.28105 (16)	0.0282 (8)	
H16	0.321116	0.475127	0.242154	0.034*	
C17	0.30710 (12)	0.6115 (7)	0.31990 (16)	0.0291 (9)	
H17	0.293684	0.750470	0.306878	0.035*	
C18	0.31060 (11)	0.5705 (6)	0.37800 (15)	0.0249 (8)	
H18	0.299935	0.683511	0.403522	0.030*	
C19	0.25933 (11)	0.4412 (6)	0.61723 (14)	0.0226 (8)	
C20	0.26414 (11)	0.6213 (6)	0.65876 (14)	0.0240 (8)	
C21	0.22874 (12)	0.7743 (6)	0.66946 (15)	0.0252 (8)	
H21	0.232343	0.897102	0.695611	0.030*	
C22	0.18814 (12)	0.7477 (6)	0.64199 (15)	0.0249 (8)	
C23	0.18351 (11)	0.5641 (6)	0.60303 (15)	0.0248 (8)	
H23	0.156118	0.542375	0.585313	0.030*	
C24	0.21846 (12)	0.4107 (6)	0.58945 (14)	0.0232 (8)	
C25	0.30762 (12)	0.6478 (7)	0.69049 (16)	0.0324 (9)	
H25A	0.312022	0.512148	0.714713	0.049*	
H25B	0.306771	0.789870	0.713210	0.049*	
H25C	0.331689	0.658303	0.663719	0.049*	
C26	0.14909 (12)	0.9077 (7)	0.65542 (17)	0.0334 (9)	
H26A	0.132792	0.843321	0.687025	0.050*	
H26B	0.129912	0.917565	0.622806	0.050*	
H26C	0.159736	1.063905	0.664950	0.050*	
C27	0.21031 (12)	0.2158 (7)	0.54676 (16)	0.0297 (9)	
H27A	0.217207	0.272939	0.509273	0.044*	
H27B	0.179610	0.168474	0.548256	0.044*	
H27C	0.228897	0.081633	0.555521	0.044*	
C28	0.51786 (11)	0.0968 (6)	0.46548 (15)	0.0224 (8)	
C29	0.51122 (12)	0.1276 (6)	0.52183 (15)	0.0239 (8)	
H29	0.490034	0.047263	0.543401	0.029*	
C30	0.54251 (11)	0.3073 (6)	0.54415 (15)	0.0218 (8)	
C31	0.56796 (12)	0.3832 (6)	0.50028 (16)	0.0225 (7)	
H31	0.589866	0.499884	0.502582	0.027*	
C32	0.55572 (11)	0.2536 (6)	0.44875 (15)	0.0229 (8)	
C33	0.57550 (11)	0.2587 (6)	0.39746 (15)	0.0243 (8)	
H33	0.564527	0.155088	0.369957	0.029*	
C34	0.49026 (11)	−0.0516 (6)	0.42789 (15)	0.0223 (8)	
C35	0.47587 (11)	0.0308 (6)	0.37476 (15)	0.0246 (8)	
H35	0.486290	0.175537	0.360834	0.030*	
C36	0.44609 (12)	−0.1027 (6)	0.34279 (16)	0.0278 (9)	
H36	0.436917	−0.047288	0.307383	0.033*	
C37	0.42992 (12)	−0.3171 (7)	0.36296 (16)	0.0297 (9)	
H37	0.409495	−0.403887	0.341633	0.036*	
C38	0.44434 (12)	−0.4018 (7)	0.41514 (17)	0.0286 (9)	
H38	0.433815	−0.546723	0.428766	0.034*	
C39	0.47439 (11)	−0.2711 (6)	0.44707 (16)	0.0245 (8)	
H39	0.484165	−0.330463	0.481852	0.029*	
C40	0.54362 (11)	0.3803 (6)	0.60410 (15)	0.0217 (8)	
C41	0.56288 (11)	0.5974 (7)	0.62138 (15)	0.0248 (8)	
H41	0.574388	0.700735	0.594173	0.030*	
C42	0.56484 (12)	0.6580 (7)	0.67802 (16)	0.0267 (9)	
H42	0.578045	0.800994	0.688734	0.032*	
C43	0.54749 (12)	0.5096 (6)	0.71904 (16)	0.0278 (9)	
H43	0.548967	0.551985	0.757264	0.033*	
C44	0.52781 (11)	0.2968 (6)	0.70301 (15)	0.0253 (8)	
H44	0.516058	0.195981	0.730586	0.030*	
C45	0.52556 (11)	0.2335 (6)	0.64608 (15)	0.0234 (8)	
H45	0.511850	0.091446	0.635730	0.028*	
C46	0.61314 (11)	0.4139 (6)	0.38060 (14)	0.0223 (8)	
C47	0.65574 (11)	0.3931 (6)	0.40497 (14)	0.0241 (8)	
C48	0.68915 (12)	0.5477 (6)	0.38677 (16)	0.0278 (9)	
H48	0.717337	0.534982	0.402891	0.033*	
C49	0.68188 (12)	0.7189 (7)	0.34564 (16)	0.0280 (9)	
C50	0.63963 (12)	0.7337 (7)	0.32137 (16)	0.0293 (9)	
H50	0.634253	0.847371	0.293421	0.035*	
C51	0.60570 (12)	0.5842 (7)	0.33773 (15)	0.0269 (8)	
C52	0.66681 (12)	0.2043 (7)	0.44841 (16)	0.0309 (9)	
H52A	0.657021	0.255505	0.485335	0.046*	
H52B	0.698413	0.178763	0.449049	0.046*	
H52C	0.652035	0.058469	0.438490	0.046*	
C53	0.71847 (13)	0.8856 (7)	0.32675 (19)	0.0387 (10)	
H53A	0.743758	0.867604	0.351372	0.058*	
H53B	0.707971	1.046960	0.328511	0.058*	
H53C	0.726934	0.848305	0.288375	0.058*	
C54	0.56037 (13)	0.6030 (8)	0.31041 (18)	0.0379 (11)	
H54A	0.553643	0.457151	0.290898	0.057*	
H54B	0.560283	0.732458	0.283720	0.057*	
H54C	0.538341	0.631357	0.339236	0.057*	

### {3-[(2,4,6-Trimethylphenyl)methylidene]-4-phenylcyclopenta-1,4-dien-1-yl}benzene (III) Atomic displacement parameters (Å^2^)

**Table:**

	*U*^11^	*U*^22^	*U*^33^	*U*^12^	*U*^13^	*U*^23^
C1	0.0209 (18)	0.0218 (18)	0.024 (2)	0.0005 (15)	0.0000 (15)	0.0015 (15)
C2	0.0235 (19)	0.0217 (19)	0.026 (2)	0.0025 (15)	0.0021 (16)	−0.0005 (15)
C3	0.0194 (18)	0.0227 (18)	0.024 (2)	0.0005 (15)	0.0014 (15)	0.0014 (15)
C4	0.0218 (18)	0.0215 (17)	0.026 (2)	0.0006 (15)	0.0007 (15)	0.0027 (15)
C5	0.0232 (18)	0.0238 (18)	0.021 (2)	−0.0012 (15)	0.0015 (15)	−0.0014 (15)
C6	0.025 (2)	0.0218 (18)	0.024 (2)	0.0012 (15)	0.0000 (15)	0.0042 (15)
C7	0.0178 (18)	0.0255 (18)	0.024 (2)	−0.0006 (14)	0.0032 (15)	0.0024 (16)
C8	0.0217 (19)	0.0255 (19)	0.026 (2)	−0.0013 (16)	0.0035 (15)	−0.0010 (15)
C9	0.0249 (19)	0.033 (2)	0.024 (2)	−0.0015 (17)	−0.0045 (16)	0.0000 (16)
C10	0.023 (2)	0.028 (2)	0.034 (2)	−0.0005 (16)	−0.0048 (16)	0.0067 (18)
C11	0.0246 (19)	0.0224 (19)	0.033 (2)	0.0018 (15)	0.0059 (17)	0.0008 (16)
C12	0.0218 (18)	0.0229 (18)	0.0233 (19)	−0.0002 (15)	0.0012 (15)	0.0013 (14)
C13	0.0159 (18)	0.0250 (18)	0.024 (2)	−0.0023 (14)	0.0020 (14)	0.0008 (14)
C14	0.022 (2)	0.0250 (18)	0.026 (2)	0.0012 (16)	0.0016 (15)	0.0008 (15)
C15	0.0273 (19)	0.030 (2)	0.028 (2)	0.0013 (16)	0.0027 (16)	−0.0023 (16)
C16	0.030 (2)	0.033 (2)	0.022 (2)	−0.0028 (17)	0.0019 (16)	0.0058 (16)
C17	0.027 (2)	0.031 (2)	0.029 (2)	0.0029 (17)	−0.0010 (16)	0.0074 (17)
C18	0.0204 (18)	0.0273 (19)	0.027 (2)	0.0004 (16)	0.0011 (15)	0.0026 (16)
C19	0.0205 (18)	0.0255 (19)	0.0218 (19)	0.0006 (15)	0.0038 (14)	0.0049 (15)
C20	0.0234 (19)	0.0287 (19)	0.020 (2)	−0.0038 (15)	0.0025 (15)	0.0024 (15)
C21	0.029 (2)	0.0262 (19)	0.0208 (19)	−0.0031 (16)	0.0045 (16)	−0.0011 (15)
C22	0.0245 (19)	0.0249 (19)	0.025 (2)	0.0004 (16)	0.0049 (16)	0.0018 (15)
C23	0.0200 (18)	0.0301 (19)	0.024 (2)	−0.0034 (15)	−0.0018 (15)	0.0033 (16)
C24	0.0276 (19)	0.0245 (18)	0.0176 (19)	−0.0023 (15)	0.0012 (15)	0.0029 (15)
C25	0.028 (2)	0.038 (2)	0.031 (2)	−0.0038 (18)	0.0002 (17)	−0.0023 (18)
C26	0.028 (2)	0.034 (2)	0.038 (2)	0.0049 (18)	0.0055 (18)	−0.0015 (18)
C27	0.027 (2)	0.035 (2)	0.027 (2)	−0.0030 (17)	−0.0005 (17)	−0.0029 (17)
C28	0.0218 (18)	0.0222 (18)	0.023 (2)	−0.0003 (15)	−0.0001 (15)	−0.0016 (15)
C29	0.0216 (19)	0.0259 (19)	0.024 (2)	−0.0019 (15)	0.0032 (15)	0.0028 (15)
C30	0.0209 (19)	0.0213 (18)	0.023 (2)	−0.0001 (15)	−0.0021 (15)	−0.0006 (15)
C31	0.0212 (18)	0.0220 (16)	0.0242 (19)	−0.0022 (16)	−0.0033 (14)	−0.0014 (17)
C32	0.0203 (18)	0.0241 (19)	0.024 (2)	0.0014 (15)	−0.0003 (15)	0.0011 (14)
C33	0.0201 (19)	0.031 (2)	0.022 (2)	0.0037 (16)	−0.0020 (15)	−0.0030 (16)
C34	0.0201 (18)	0.0234 (18)	0.0235 (19)	0.0018 (15)	0.0029 (15)	−0.0035 (15)
C35	0.0213 (19)	0.0252 (18)	0.027 (2)	0.0003 (15)	0.0023 (15)	−0.0038 (16)
C36	0.0229 (19)	0.035 (2)	0.025 (2)	0.0047 (17)	0.0005 (16)	−0.0074 (16)
C37	0.0205 (19)	0.035 (2)	0.034 (2)	−0.0042 (17)	0.0019 (16)	−0.0136 (18)
C38	0.0235 (19)	0.025 (2)	0.038 (2)	−0.0033 (16)	0.0050 (17)	−0.0067 (17)
C39	0.0211 (18)	0.028 (2)	0.024 (2)	−0.0001 (15)	0.0024 (15)	−0.0016 (15)
C40	0.0195 (18)	0.0255 (19)	0.0200 (19)	0.0028 (15)	−0.0020 (15)	−0.0003 (15)
C41	0.0234 (19)	0.0266 (19)	0.024 (2)	−0.0042 (15)	0.0003 (15)	0.0033 (15)
C42	0.025 (2)	0.027 (2)	0.028 (2)	−0.0017 (16)	−0.0009 (16)	−0.0023 (16)
C43	0.025 (2)	0.037 (2)	0.022 (2)	0.0038 (17)	−0.0031 (15)	−0.0020 (17)
C44	0.0213 (18)	0.034 (2)	0.020 (2)	0.0024 (16)	0.0010 (15)	0.0040 (16)
C45	0.0186 (19)	0.0277 (19)	0.024 (2)	−0.0007 (15)	−0.0008 (15)	0.0010 (16)
C46	0.0243 (19)	0.0253 (19)	0.0172 (19)	−0.0003 (15)	0.0020 (15)	−0.0018 (15)
C47	0.0215 (19)	0.028 (2)	0.023 (2)	0.0029 (16)	0.0035 (15)	0.0007 (15)
C48	0.0195 (19)	0.031 (2)	0.033 (2)	0.0033 (16)	0.0000 (16)	−0.0014 (17)
C49	0.023 (2)	0.028 (2)	0.032 (2)	0.0012 (16)	0.0062 (16)	0.0000 (17)
C50	0.028 (2)	0.031 (2)	0.028 (2)	0.0054 (17)	0.0064 (16)	0.0085 (16)
C51	0.0230 (19)	0.034 (2)	0.024 (2)	0.0043 (16)	0.0009 (15)	0.0014 (16)
C52	0.025 (2)	0.035 (2)	0.032 (2)	0.0035 (17)	−0.0031 (17)	0.0060 (17)
C53	0.033 (2)	0.035 (2)	0.047 (3)	−0.0041 (18)	0.0066 (19)	0.009 (2)
C54	0.028 (2)	0.049 (3)	0.037 (3)	−0.0001 (19)	−0.0065 (18)	0.014 (2)

### {3-[(2,4,6-Trimethylphenyl)methylidene]-4-phenylcyclopenta-1,4-dien-1-yl}benzene (III) Geometric parameters (Å, º)

**Table:**

C1—C2	1.354 (5)	C28—C29	1.350 (5)
C1—C5	1.476 (5)	C28—C32	1.491 (5)
C1—C7	1.474 (5)	C28—C34	1.470 (5)
C2—H2	0.9300	C29—H29	0.9300
C2—C3	1.473 (5)	C29—C30	1.475 (5)
C3—C4	1.352 (5)	C30—C31	1.352 (5)
C3—C13	1.466 (5)	C30—C40	1.467 (5)
C4—H4	0.9300	C31—H31	0.9300
C4—C5	1.461 (5)	C31—C32	1.460 (5)
C5—C6	1.351 (5)	C32—C33	1.344 (5)
C6—H6	0.9300	C33—H33	0.9300
C6—C19	1.478 (5)	C33—C46	1.481 (5)
C7—C8	1.405 (5)	C34—C35	1.400 (5)
C7—C12	1.400 (5)	C34—C39	1.396 (5)
C8—H8	0.9300	C35—H35	0.9300
C8—C9	1.381 (5)	C35—C36	1.388 (5)
C9—H9	0.9300	C36—H36	0.9300
C9—C10	1.377 (5)	C36—C37	1.382 (5)
C10—H10	0.9300	C37—H37	0.9300
C10—C11	1.382 (6)	C37—C38	1.384 (5)
C11—H11	0.9300	C38—H38	0.9300
C11—C12	1.381 (5)	C38—C39	1.384 (5)
C12—H12	0.9300	C39—H39	0.9300
C13—C14	1.403 (5)	C40—C41	1.409 (5)
C13—C18	1.399 (5)	C40—C45	1.395 (5)
C14—H14	0.9300	C41—H41	0.9300
C14—C15	1.382 (5)	C41—C42	1.375 (5)
C15—H15	0.9300	C42—H42	0.9300
C15—C16	1.380 (5)	C42—C43	1.376 (5)
C16—H16	0.9300	C43—H43	0.9300
C16—C17	1.388 (5)	C43—C44	1.385 (5)
C17—H17	0.9300	C44—H44	0.9300
C17—C18	1.388 (5)	C44—C45	1.386 (5)
C18—H18	0.9300	C45—H45	0.9300
C19—C20	1.412 (5)	C46—C47	1.407 (5)
C19—C24	1.401 (5)	C46—C51	1.407 (5)
C20—C21	1.390 (5)	C47—C48	1.394 (5)
C20—C25	1.511 (5)	C47—C52	1.509 (5)
C21—H21	0.9300	C48—H48	0.9300
C21—C22	1.388 (5)	C48—C49	1.381 (5)
C22—C23	1.386 (5)	C49—C50	1.393 (5)
C22—C26	1.510 (5)	C49—C53	1.510 (5)
C23—H23	0.9300	C50—H50	0.9300
C23—C24	1.395 (5)	C50—C51	1.375 (5)
C24—C27	1.505 (5)	C51—C54	1.509 (5)
C25—H25A	0.9600	C52—H52A	0.9600
C25—H25B	0.9600	C52—H52B	0.9600
C25—H25C	0.9600	C52—H52C	0.9600
C26—H26A	0.9600	C53—H53A	0.9600
C26—H26B	0.9600	C53—H53B	0.9600
C26—H26C	0.9600	C53—H53C	0.9600
C27—H27A	0.9600	C54—H54A	0.9600
C27—H27B	0.9600	C54—H54B	0.9600
C27—H27C	0.9600	C54—H54C	0.9600
			
C2—C1—C5	107.1 (3)	C29—C28—C32	107.2 (3)
C2—C1—C7	125.5 (3)	C29—C28—C34	125.3 (3)
C7—C1—C5	127.4 (3)	C34—C28—C32	127.3 (3)
C1—C2—H2	125.1	C28—C29—H29	125.0
C1—C2—C3	109.8 (3)	C28—C29—C30	110.0 (3)
C3—C2—H2	125.1	C30—C29—H29	125.0
C4—C3—C2	108.0 (3)	C31—C30—C29	107.7 (3)
C4—C3—C13	128.1 (3)	C31—C30—C40	129.1 (3)
C13—C3—C2	123.9 (3)	C40—C30—C29	123.1 (3)
C3—C4—H4	125.6	C30—C31—H31	125.3
C3—C4—C5	108.7 (3)	C30—C31—C32	109.4 (3)
C5—C4—H4	125.6	C32—C31—H31	125.3
C4—C5—C1	106.2 (3)	C31—C32—C28	105.5 (3)
C6—C5—C1	126.8 (3)	C33—C32—C28	125.9 (3)
C6—C5—C4	126.6 (3)	C33—C32—C31	128.4 (3)
C5—C6—H6	117.3	C32—C33—H33	116.9
C5—C6—C19	125.3 (3)	C32—C33—C46	126.2 (3)
C19—C6—H6	117.3	C46—C33—H33	116.9
C8—C7—C1	122.4 (3)	C35—C34—C28	121.5 (3)
C12—C7—C1	119.6 (3)	C39—C34—C28	119.9 (3)
C12—C7—C8	117.9 (3)	C39—C34—C35	118.3 (3)
C7—C8—H8	119.7	C34—C35—H35	119.9
C9—C8—C7	120.6 (3)	C36—C35—C34	120.2 (3)
C9—C8—H8	119.7	C36—C35—H35	119.9
C8—C9—H9	119.8	C35—C36—H36	119.6
C10—C9—C8	120.5 (4)	C37—C36—C35	120.8 (4)
C10—C9—H9	119.8	C37—C36—H36	119.6
C9—C10—H10	120.1	C36—C37—H37	120.2
C9—C10—C11	119.8 (4)	C36—C37—C38	119.5 (3)
C11—C10—H10	120.1	C38—C37—H37	120.2
C10—C11—H11	119.8	C37—C38—H38	119.9
C12—C11—C10	120.3 (3)	C39—C38—C37	120.1 (4)
C12—C11—H11	119.8	C39—C38—H38	119.9
C7—C12—H12	119.6	C34—C39—H39	119.5
C11—C12—C7	120.8 (3)	C38—C39—C34	121.0 (3)
C11—C12—H12	119.6	C38—C39—H39	119.5
C14—C13—C3	119.7 (3)	C41—C40—C30	121.8 (3)
C18—C13—C3	122.5 (3)	C45—C40—C30	120.3 (3)
C18—C13—C14	117.8 (3)	C45—C40—C41	117.8 (3)
C13—C14—H14	119.5	C40—C41—H41	119.6
C15—C14—C13	121.0 (3)	C42—C41—C40	120.7 (3)
C15—C14—H14	119.5	C42—C41—H41	119.6
C14—C15—H15	119.7	C41—C42—H42	119.6
C16—C15—C14	120.7 (4)	C41—C42—C43	120.8 (3)
C16—C15—H15	119.7	C43—C42—H42	119.6
C15—C16—H16	120.4	C42—C43—H43	120.2
C15—C16—C17	119.2 (4)	C42—C43—C44	119.6 (3)
C17—C16—H16	120.4	C44—C43—H43	120.2
C16—C17—H17	119.7	C43—C44—H44	119.8
C16—C17—C18	120.6 (3)	C43—C44—C45	120.3 (3)
C18—C17—H17	119.7	C45—C44—H44	119.8
C13—C18—H18	119.6	C40—C45—H45	119.6
C17—C18—C13	120.7 (3)	C44—C45—C40	120.8 (3)
C17—C18—H18	119.6	C44—C45—H45	119.6
C20—C19—C6	117.7 (3)	C47—C46—C33	122.4 (3)
C24—C19—C6	122.4 (3)	C47—C46—C51	119.5 (3)
C24—C19—C20	119.9 (3)	C51—C46—C33	118.1 (3)
C19—C20—C25	120.0 (3)	C46—C47—C52	122.3 (3)
C21—C20—C19	119.3 (3)	C48—C47—C46	118.6 (3)
C21—C20—C25	120.7 (3)	C48—C47—C52	119.1 (3)
C20—C21—H21	119.3	C47—C48—H48	118.8
C22—C21—C20	121.5 (3)	C49—C48—C47	122.3 (3)
C22—C21—H21	119.3	C49—C48—H48	118.8
C21—C22—C26	121.4 (3)	C48—C49—C50	118.2 (3)
C23—C22—C21	118.4 (3)	C48—C49—C53	121.5 (3)
C23—C22—C26	120.2 (3)	C50—C49—C53	120.4 (3)
C22—C23—H23	118.8	C49—C50—H50	119.2
C22—C23—C24	122.4 (3)	C51—C50—C49	121.6 (3)
C24—C23—H23	118.8	C51—C50—H50	119.2
C19—C24—C27	122.8 (3)	C46—C51—C54	119.7 (3)
C23—C24—C19	118.5 (3)	C50—C51—C46	119.8 (3)
C23—C24—C27	118.6 (3)	C50—C51—C54	120.5 (3)
C20—C25—H25A	109.5	C47—C52—H52A	109.5
C20—C25—H25B	109.5	C47—C52—H52B	109.5
C20—C25—H25C	109.5	C47—C52—H52C	109.5
H25A—C25—H25B	109.5	H52A—C52—H52B	109.5
H25A—C25—H25C	109.5	H52A—C52—H52C	109.5
H25B—C25—H25C	109.5	H52B—C52—H52C	109.5
C22—C26—H26A	109.5	C49—C53—H53A	109.5
C22—C26—H26B	109.5	C49—C53—H53B	109.5
C22—C26—H26C	109.5	C49—C53—H53C	109.5
H26A—C26—H26B	109.5	H53A—C53—H53B	109.5
H26A—C26—H26C	109.5	H53A—C53—H53C	109.5
H26B—C26—H26C	109.5	H53B—C53—H53C	109.5
C24—C27—H27A	109.5	C51—C54—H54A	109.5
C24—C27—H27B	109.5	C51—C54—H54B	109.5
C24—C27—H27C	109.5	C51—C54—H54C	109.5
H27A—C27—H27B	109.5	H54A—C54—H54B	109.5
H27A—C27—H27C	109.5	H54A—C54—H54C	109.5
H27B—C27—H27C	109.5	H54B—C54—H54C	109.5

### {3-[(2,4,6-Trimethylphenyl)methylidene]-4-phenylcyclopenta-1,4-dien-1-yl}benzene (III) Hydrogen-bond geometry (Å, º)

*Cg*5, *Cg*6, *Cg*7, *Cg*8, and *Cg*9 are the centroids of the C40–C45, C7–C12, C13–C18, C28–C32, and C34–C39 rings, respectively.

**Table:**

*D*—H···*A*	*D*—H	H···*A*	*D*···*A*	*D*—H···*A*
C10—H10···*Cg*5^i^	0.93	2.81	3.5059 (7)	132
C25—H25*C*···*Cg*6^ii^	0.96	2.81	3.7115 (8)	158
C37—H37···*Cg*7^iii^	0.93	2.71	3.5129 (8)	145
C39—H39···*Cg*8^ii^	0.93	2.97	3.5817 (8)	125
C53—H53*A*···*Cg*7^iv^	0.96	2.94	3.6503 (8)	132
C54—H54*C*···*Cg*9^v^	0.96	2.88	3.7983 (8)	160

Symmetry codes: (i) −*x*, −*y*, *z*−1/2; (ii) *x*, *y*+1, *z*; (iii) −*x*, −*y*+2, *z*+1/2; (iv) *x*+1/2, −*y*, *z*; (v) *x*, *y*−1, *z*.

### {3-[(2,3,4,5,6-Pentamethylphenyl)methylidene]-4-phenylcyclopenta-1,4-dien-1-yl}benzene (IV) Crystal data

**Table:**

C_29_H_28_	*F*(000) = 1616
*M**_r_* = 376.51	*D*_x_ = 1.183 Mg m^−^^3^
Monoclinic, *C*2/*c*	Mo *K*α radiation, λ = 0.71073 Å
*a* = 30.987 (7) Å	Cell parameters from 3475 reflections
*b* = 5.8273 (14) Å	θ = 2.3–26.2°
*c* = 23.557 (6) Å	µ = 0.07 mm^−^^1^
β = 96.192 (3)°	*T* = 100 K
*V* = 4228.9 (17) Å^3^	Needle, orange
*Z* = 8	0.44 × 0.11 × 0.1 mm

### {3-[(2,3,4,5,6-Pentamethylphenyl)methylidene]-4-phenylcyclopenta-1,4-dien-1-yl}benzene (IV) Data collection

**Table:**

Bruker APEXII CCD diffractometer	3213 reflections with *I* > 2σ(*I*)
φ and ω scans	*R*_int_ = 0.082
Absorption correction: multi-scan SADABS	θ_max_ = 26.5°, θ_min_ = 2.1°
*T*_min_ = 0.587, *T*_max_ = 0.745	*h* = −38→38
37965 measured reflections	*k* = −7→7
4394 independent reflections	*l* = −29→29

### {3-[(2,3,4,5,6-Pentamethylphenyl)methylidene]-4-phenylcyclopenta-1,4-dien-1-yl}benzene (IV) Refinement

**Table:**

Refinement on *F*^2^	Primary atom site location: dual
Least-squares matrix: full	Hydrogen site location: inferred from neighbouring sites
*R*[*F*^2^ > 2σ(*F*^2^)] = 0.050	H-atom parameters constrained
*wR*(*F*^2^) = 0.137	*w* = 1/[σ^2^(*F*_o_^2^) + (0.0562*P*)^2^ + 4.4117*P*] where *P* = (*F*_o_^2^ + 2*F*_c_^2^)/3
*S* = 1.03	(Δ/σ)_max_ = 0.001
4394 reflections	Δρ_max_ = 0.24 e Å^−^^3^
267 parameters	Δρ_min_ = −0.22 e Å^−^^3^
0 restraints	

### {3-[(2,3,4,5,6-Pentamethylphenyl)methylidene]-4-phenylcyclopenta-1,4-dien-1-yl}benzene (IV) Special details

**Table:**

Geometry. All esds (except the esd in the dihedral angle between two l.s. planes) are estimated using the full covariance matrix. The cell esds are taken into account individually in the estimation of esds in distances, angles and torsion angles; correlations between esds in cell parameters are only used when they are defined by crystal symmetry. An approximate (isotropic) treatment of cell esds is used for estimating esds involving l.s. planes.

### {3-[(2,3,4,5,6-Pentamethylphenyl)methylidene]-4-phenylcyclopenta-1,4-dien-1-yl}benzene (IV) Fractional atomic coordinates and isotropic or equivalent isotropic displacement parameters (Å^2^)

**Table:**

	*x*	*y*	*z*	*U*_iso_*/*U*_eq_	
C13	0.59242 (5)	0.5116 (3)	0.64414 (7)	0.0214 (4)	
C7	0.55646 (5)	0.0887 (3)	0.82183 (7)	0.0213 (4)	
C19	0.67937 (5)	0.5436 (3)	0.86724 (7)	0.0214 (4)	
C5	0.61684 (5)	0.3893 (3)	0.79995 (7)	0.0221 (4)	
C20	0.67537 (6)	0.7194 (3)	0.90741 (7)	0.0221 (4)	
C4	0.62469 (6)	0.5125 (3)	0.74821 (7)	0.0228 (4)	
H4	0.646359	0.626148	0.745542	0.027*	
C14	0.57411 (6)	0.3657 (3)	0.60104 (7)	0.0242 (4)	
H14	0.563145	0.220269	0.610728	0.029*	
C12	0.54703 (5)	0.1649 (3)	0.87570 (7)	0.0229 (4)	
H12	0.556500	0.312444	0.888929	0.027*	
C8	0.54204 (5)	−0.1290 (3)	0.80385 (7)	0.0233 (4)	
H8	0.548662	−0.185405	0.767949	0.028*	
C3	0.59587 (5)	0.4381 (3)	0.70428 (7)	0.0216 (4)	
C1	0.57895 (5)	0.2366 (3)	0.78360 (7)	0.0222 (4)	
C2	0.56767 (5)	0.2650 (3)	0.72698 (7)	0.0221 (4)	
H2	0.544904	0.185041	0.705081	0.026*	
C18	0.60730 (6)	0.7271 (3)	0.62895 (7)	0.0241 (4)	
H18	0.618881	0.830511	0.657840	0.029*	
C6	0.64079 (6)	0.3964 (3)	0.85109 (7)	0.0230 (4)	
H6	0.632173	0.297150	0.879814	0.028*	
C24	0.71917 (6)	0.5018 (3)	0.84579 (7)	0.0247 (4)	
C23	0.75536 (6)	0.6403 (3)	0.86477 (7)	0.0259 (4)	
C10	0.50940 (6)	−0.1886 (3)	0.89086 (8)	0.0273 (4)	
H10	0.493552	−0.282816	0.914192	0.033*	
C11	0.52397 (6)	0.0264 (3)	0.90983 (7)	0.0257 (4)	
H11	0.518138	0.079165	0.946387	0.031*	
C16	0.58769 (6)	0.6439 (3)	0.52940 (7)	0.0279 (4)	
H16	0.586508	0.687795	0.490425	0.033*	
C21	0.71147 (6)	0.8557 (3)	0.92605 (7)	0.0241 (4)	
C9	0.51823 (6)	−0.2643 (3)	0.83749 (8)	0.0263 (4)	
H9	0.507883	−0.409838	0.823913	0.032*	
C22	0.75145 (6)	0.8155 (3)	0.90470 (7)	0.0258 (4)	
C15	0.57181 (6)	0.4318 (3)	0.54404 (7)	0.0273 (4)	
H15	0.559312	0.331309	0.515037	0.033*	
C25	0.63232 (6)	0.7587 (3)	0.93028 (8)	0.0274 (4)	
H25A	0.634909	0.720712	0.971033	0.041*	
H25B	0.610176	0.660797	0.909650	0.041*	
H25C	0.623921	0.920034	0.925004	0.041*	
C17	0.60526 (6)	0.7913 (3)	0.57185 (7)	0.0262 (4)	
H17	0.615984	0.936980	0.561929	0.031*	
C29	0.72431 (6)	0.3065 (4)	0.80483 (8)	0.0315 (5)	
H29A	0.697383	0.217050	0.799632	0.047*	
H29B	0.748259	0.207275	0.820370	0.047*	
H29C	0.730603	0.368437	0.767938	0.047*	
C26	0.70788 (6)	1.0445 (3)	0.96918 (8)	0.0323 (5)	
H26A	0.728286	1.014941	1.003141	0.048*	
H26B	0.678224	1.049016	0.979974	0.048*	
H26C	0.714808	1.192160	0.952417	0.048*	
C28	0.79882 (6)	0.5958 (4)	0.84287 (8)	0.0361 (5)	
H28A	0.810760	0.740673	0.830322	0.054*	
H28B	0.794943	0.488770	0.810634	0.054*	
H28C	0.818874	0.529184	0.873492	0.054*	
C27	0.79006 (6)	0.9625 (4)	0.92679 (8)	0.0353 (5)	
H27A	0.796079	0.942774	0.968195	0.053*	
H27B	0.783513	1.124120	0.918115	0.053*	
H27C	0.815519	0.915727	0.908311	0.053*	

### {3-[(2,3,4,5,6-Pentamethylphenyl)methylidene]-4-phenylcyclopenta-1,4-dien-1-yl}benzene (IV) Atomic displacement parameters (Å^2^)

**Table:**

	*U*^11^	*U*^22^	*U*^33^	*U*^12^	*U*^13^	*U*^23^
C13	0.0167 (8)	0.0278 (10)	0.0198 (8)	0.0020 (7)	0.0020 (7)	0.0000 (7)
C7	0.0150 (8)	0.0271 (9)	0.0210 (8)	−0.0010 (7)	−0.0020 (7)	0.0029 (7)
C19	0.0204 (9)	0.0265 (9)	0.0164 (8)	−0.0024 (7)	−0.0016 (7)	0.0039 (7)
C5	0.0197 (9)	0.0242 (9)	0.0222 (9)	−0.0013 (7)	0.0009 (7)	−0.0007 (7)
C20	0.0216 (9)	0.0257 (9)	0.0182 (8)	−0.0012 (7)	−0.0015 (7)	0.0046 (7)
C4	0.0203 (9)	0.0263 (9)	0.0215 (9)	−0.0013 (7)	0.0019 (7)	0.0020 (7)
C14	0.0212 (9)	0.0273 (10)	0.0242 (9)	0.0018 (7)	0.0027 (7)	−0.0006 (7)
C12	0.0196 (9)	0.0258 (9)	0.0224 (9)	−0.0016 (7)	−0.0022 (7)	0.0006 (7)
C8	0.0198 (9)	0.0264 (10)	0.0232 (9)	0.0009 (7)	−0.0003 (7)	0.0012 (7)
C3	0.0205 (9)	0.0229 (9)	0.0216 (9)	0.0015 (7)	0.0033 (7)	0.0004 (7)
C1	0.0205 (9)	0.0250 (9)	0.0208 (9)	−0.0005 (7)	0.0009 (7)	0.0000 (7)
C2	0.0190 (9)	0.0258 (9)	0.0210 (8)	−0.0018 (7)	0.0000 (7)	−0.0019 (7)
C18	0.0225 (9)	0.0275 (10)	0.0219 (9)	0.0007 (8)	0.0011 (7)	−0.0005 (7)
C6	0.0228 (9)	0.0257 (9)	0.0204 (8)	−0.0028 (7)	0.0018 (7)	0.0021 (7)
C24	0.0240 (9)	0.0301 (10)	0.0191 (8)	0.0002 (8)	−0.0016 (7)	0.0044 (7)
C23	0.0197 (9)	0.0344 (11)	0.0231 (9)	−0.0015 (8)	−0.0004 (7)	0.0067 (8)
C10	0.0195 (9)	0.0336 (11)	0.0283 (10)	−0.0031 (8)	0.0002 (7)	0.0079 (8)
C11	0.0204 (9)	0.0350 (11)	0.0217 (9)	0.0010 (8)	0.0015 (7)	0.0013 (8)
C16	0.0276 (10)	0.0373 (11)	0.0190 (9)	0.0081 (8)	0.0035 (7)	0.0040 (8)
C21	0.0245 (9)	0.0261 (10)	0.0206 (8)	−0.0023 (8)	−0.0025 (7)	0.0043 (7)
C9	0.0227 (9)	0.0238 (10)	0.0312 (10)	−0.0034 (7)	−0.0033 (8)	0.0028 (8)
C22	0.0207 (9)	0.0316 (10)	0.0238 (9)	−0.0042 (8)	−0.0037 (7)	0.0069 (8)
C15	0.0245 (9)	0.0362 (11)	0.0208 (9)	0.0040 (8)	0.0001 (7)	−0.0049 (8)
C25	0.0211 (9)	0.0343 (11)	0.0265 (9)	−0.0006 (8)	0.0012 (7)	0.0012 (8)
C17	0.0234 (9)	0.0300 (10)	0.0255 (9)	0.0028 (8)	0.0047 (7)	0.0049 (8)
C29	0.0275 (10)	0.0393 (12)	0.0274 (10)	0.0010 (9)	0.0016 (8)	−0.0027 (8)
C26	0.0303 (10)	0.0333 (11)	0.0321 (10)	−0.0053 (9)	−0.0025 (8)	−0.0029 (8)
C28	0.0234 (10)	0.0534 (14)	0.0315 (10)	0.0003 (9)	0.0026 (8)	0.0057 (10)
C27	0.0271 (10)	0.0425 (12)	0.0350 (11)	−0.0111 (9)	−0.0025 (8)	0.0037 (9)

### {3-[(2,3,4,5,6-Pentamethylphenyl)methylidene]-4-phenylcyclopenta-1,4-dien-1-yl}benzene (IV) Geometric parameters (Å, º)

**Table:**

C13—C14	1.397 (2)	C23—C22	1.402 (3)
C13—C3	1.473 (2)	C23—C28	1.516 (3)
C13—C18	1.397 (3)	C10—H10	0.9500
C7—C12	1.405 (2)	C10—C11	1.389 (3)
C7—C8	1.396 (3)	C10—C9	1.387 (3)
C7—C1	1.475 (2)	C11—H11	0.9500
C19—C20	1.409 (2)	C16—H16	0.9500
C19—C6	1.487 (2)	C16—C15	1.388 (3)
C19—C24	1.404 (2)	C16—C17	1.384 (3)
C5—C4	1.457 (2)	C21—C22	1.406 (3)
C5—C1	1.490 (2)	C21—C26	1.510 (3)
C5—C6	1.346 (2)	C9—H9	0.9500
C20—C21	1.403 (2)	C22—C27	1.517 (3)
C20—C25	1.509 (2)	C15—H15	0.9500
C4—H4	0.9500	C25—H25A	0.9800
C4—C3	1.363 (2)	C25—H25B	0.9800
C14—H14	0.9500	C25—H25C	0.9800
C14—C15	1.391 (2)	C17—H17	0.9500
C12—H12	0.9500	C29—H29A	0.9800
C12—C11	1.390 (2)	C29—H29B	0.9800
C8—H8	0.9500	C29—H29C	0.9800
C8—C9	1.386 (2)	C26—H26A	0.9800
C3—C2	1.472 (2)	C26—H26B	0.9800
C1—C2	1.352 (2)	C26—H26C	0.9800
C2—H2	0.9500	C28—H28A	0.9800
C18—H18	0.9500	C28—H28B	0.9800
C18—C17	1.391 (2)	C28—H28C	0.9800
C6—H6	0.9500	C27—H27A	0.9800
C24—C23	1.415 (3)	C27—H27B	0.9800
C24—C29	1.511 (3)	C27—H27C	0.9800
			
C14—C13—C3	120.24 (16)	C12—C11—H11	119.8
C14—C13—C18	118.70 (16)	C10—C11—C12	120.48 (17)
C18—C13—C3	121.06 (16)	C10—C11—H11	119.8
C12—C7—C1	121.66 (16)	C15—C16—H16	120.1
C8—C7—C12	118.08 (16)	C17—C16—H16	120.1
C8—C7—C1	120.18 (16)	C17—C16—C15	119.70 (17)
C20—C19—C6	117.68 (15)	C20—C21—C22	119.81 (17)
C24—C19—C20	120.83 (16)	C20—C21—C26	120.60 (16)
C24—C19—C6	121.39 (16)	C22—C21—C26	119.59 (16)
C4—C5—C1	106.01 (14)	C8—C9—C10	120.46 (17)
C6—C5—C4	127.44 (16)	C8—C9—H9	119.8
C6—C5—C1	126.35 (16)	C10—C9—H9	119.8
C19—C20—C25	119.69 (16)	C23—C22—C21	120.30 (16)
C21—C20—C19	119.79 (16)	C23—C22—C27	121.28 (17)
C21—C20—C25	120.52 (16)	C21—C22—C27	118.42 (17)
C5—C4—H4	125.6	C14—C15—H15	119.9
C3—C4—C5	108.87 (16)	C16—C15—C14	120.25 (17)
C3—C4—H4	125.6	C16—C15—H15	119.9
C13—C14—H14	119.8	C20—C25—H25A	109.5
C15—C14—C13	120.50 (18)	C20—C25—H25B	109.5
C15—C14—H14	119.8	C20—C25—H25C	109.5
C7—C12—H12	119.7	H25A—C25—H25B	109.5
C11—C12—C7	120.59 (17)	H25A—C25—H25C	109.5
C11—C12—H12	119.7	H25B—C25—H25C	109.5
C7—C8—H8	119.5	C18—C17—H17	119.8
C9—C8—C7	121.08 (17)	C16—C17—C18	120.32 (18)
C9—C8—H8	119.5	C16—C17—H17	119.8
C4—C3—C13	127.69 (16)	C24—C29—H29A	109.5
C4—C3—C2	107.95 (15)	C24—C29—H29B	109.5
C2—C3—C13	124.37 (15)	C24—C29—H29C	109.5
C7—C1—C5	127.02 (15)	H29A—C29—H29B	109.5
C2—C1—C7	125.79 (16)	H29A—C29—H29C	109.5
C2—C1—C5	107.15 (15)	H29B—C29—H29C	109.5
C3—C2—H2	125.0	C21—C26—H26A	109.5
C1—C2—C3	109.98 (15)	C21—C26—H26B	109.5
C1—C2—H2	125.0	C21—C26—H26C	109.5
C13—C18—H18	119.8	H26A—C26—H26B	109.5
C17—C18—C13	120.49 (17)	H26A—C26—H26C	109.5
C17—C18—H18	119.8	H26B—C26—H26C	109.5
C19—C6—H6	116.7	C23—C28—H28A	109.5
C5—C6—C19	126.66 (16)	C23—C28—H28B	109.5
C5—C6—H6	116.7	C23—C28—H28C	109.5
C19—C24—C23	118.97 (17)	H28A—C28—H28B	109.5
C19—C24—C29	121.18 (16)	H28A—C28—H28C	109.5
C23—C24—C29	119.81 (16)	H28B—C28—H28C	109.5
C24—C23—C28	119.87 (17)	C22—C27—H27A	109.5
C22—C23—C24	120.31 (17)	C22—C27—H27B	109.5
C22—C23—C28	119.81 (17)	C22—C27—H27C	109.5
C11—C10—H10	120.4	H27A—C27—H27B	109.5
C9—C10—H10	120.4	H27A—C27—H27C	109.5
C9—C10—C11	119.27 (17)	H27B—C27—H27C	109.5
			
C13—C14—C15—C16	−0.1 (3)	C3—C13—C18—C17	177.71 (16)
C13—C3—C2—C1	−179.76 (16)	C1—C7—C12—C11	176.90 (16)
C13—C18—C17—C16	1.2 (3)	C1—C7—C8—C9	−175.49 (16)
C7—C12—C11—C10	−0.8 (3)	C1—C5—C4—C3	−1.99 (19)
C7—C8—C9—C10	−2.1 (3)	C1—C5—C6—C19	178.18 (17)
C7—C1—C2—C3	175.98 (16)	C18—C13—C14—C15	1.7 (3)
C19—C20—C21—C22	−0.3 (2)	C18—C13—C3—C4	−25.0 (3)
C19—C20—C21—C26	−179.85 (16)	C18—C13—C3—C2	155.29 (17)
C19—C24—C23—C22	0.4 (3)	C6—C19—C20—C21	176.97 (15)
C19—C24—C23—C28	179.05 (16)	C6—C19—C20—C25	−2.7 (2)
C5—C4—C3—C13	−178.73 (16)	C6—C19—C24—C23	−176.92 (16)
C5—C4—C3—C2	1.0 (2)	C6—C19—C24—C29	0.7 (3)
C5—C1—C2—C3	−1.7 (2)	C6—C5—C4—C3	173.08 (18)
C20—C19—C6—C5	112.0 (2)	C6—C5—C1—C7	9.5 (3)
C20—C19—C24—C23	−0.5 (3)	C6—C5—C1—C2	−172.87 (18)
C20—C19—C24—C29	177.06 (16)	C24—C19—C20—C21	0.4 (2)
C20—C21—C22—C23	0.2 (3)	C24—C19—C20—C25	−179.27 (16)
C20—C21—C22—C27	−178.62 (16)	C24—C19—C6—C5	−71.5 (3)
C4—C5—C1—C7	−175.40 (16)	C24—C23—C22—C21	−0.3 (3)
C4—C5—C1—C2	2.27 (19)	C24—C23—C22—C27	178.50 (16)
C4—C5—C6—C19	4.1 (3)	C11—C10—C9—C8	1.2 (3)
C4—C3—C2—C1	0.5 (2)	C9—C10—C11—C12	0.2 (3)
C14—C13—C3—C4	154.94 (18)	C15—C16—C17—C18	0.4 (3)
C14—C13—C3—C2	−24.7 (3)	C25—C20—C21—C22	179.43 (16)
C14—C13—C18—C17	−2.3 (3)	C25—C20—C21—C26	−0.1 (2)
C12—C7—C8—C9	1.5 (2)	C17—C16—C15—C14	−1.0 (3)
C12—C7—C1—C5	42.2 (3)	C29—C24—C23—C22	−177.17 (16)
C12—C7—C1—C2	−135.07 (19)	C29—C24—C23—C28	1.4 (3)
C8—C7—C12—C11	0.0 (2)	C26—C21—C22—C23	179.79 (16)
C8—C7—C1—C5	−140.94 (18)	C26—C21—C22—C27	1.0 (2)
C8—C7—C1—C2	41.8 (3)	C28—C23—C22—C21	−178.90 (16)
C3—C13—C14—C15	−178.30 (16)	C28—C23—C22—C27	−0.1 (3)

### {3-[(2,3,4,5,6-Pentamethylphenyl)methylidene]-4-phenylcyclopenta-1,4-dien-1-yl}benzene (IV) Hydrogen-bond geometry (Å, º)

*Cg*10 is the centroid of the C13–C18 ring.

**Table:**

*D*—H···*A*	*D*—H	H···*A*	*D*···*A*	*D*—H···*A*
C10—H10···*Cg*10^i^	0.95	2.70	3.4521 (9)	136

Symmetry code: (i) −*x*, *y*+1, −*z*+1/2.
